# Supplementary material for: A type VII-secreted lipase toxin with reverse domain arrangement
Source: Nat Commun. 2023 Dec 19;14:8438. doi: 10.1038/s41467-023-44221-y (PMC10730906; doi:10.1038/s41467-023-44221-y)
Supplement: Supplementary file 1 — Supplementary Information [file 41467_2023_44221_MOESM1_ESM.pdf]

Supplementary Information:

A type VII-secreted lipase toxin with reverse domain arrangement

Supplementary Figures

Supplementary Fig. 1. SAOUHSC_00406/TslA has a polymorphic N-terminal region.
Supplementary Fig. 2. Two further homologues of TslA can be encoded in *S. aureus* genomes.
Supplementary Fig. 3. The split nanoluciferase assay indicates that TslA is detected in the culture supernatant when co-produced with TlaA1 and TlaA2.
Supplementary Fig. 4. The raw data for the split nanoluciferase assays presented in Fig. 1e and Supplementary Fig 3.
Supplementary Fig. 5. Bacterial two-hybrid analysis of the four proteins encoded at the *tslA* cluster.
Supplementary Fig. 6. TslA and its isolated N-terminal domain have lipase activity against the model lipid substrate Tween 20.
Supplementary Fig. 7. *S. aureus* strains encode three DUF576 tandem lipoprotein (Til1) islands and one orphan Til1.
Supplementary Fig. 8. The TilA immunity protein inhibits TslA lipase activity.
Supplementary Fig. 9. TslA and catalytically inactive variants are produced at similar levels in USA300 strains.
Supplementary Fig. 10. Uncropped Western blots.
Supplementary Fig. 11. Cryo-EM processing workflow, showing local and global map quality.

Supplementary Tables

Supplementary Table 1. Analysis of Tsl1 distribution in *S. aureus* genomes.
Supplementary Table 2. Analysis of *S. aureus* USA300 cells stained by Sytox green when imaged using fluorescence microscopy.
Supplementary Table 3. Bacterial strains used in this work.
Supplementary Table 4. Plasmids used in this work.
Supplementary Table 5. Protein expression and purification conditions used in this work.

Supplementary References

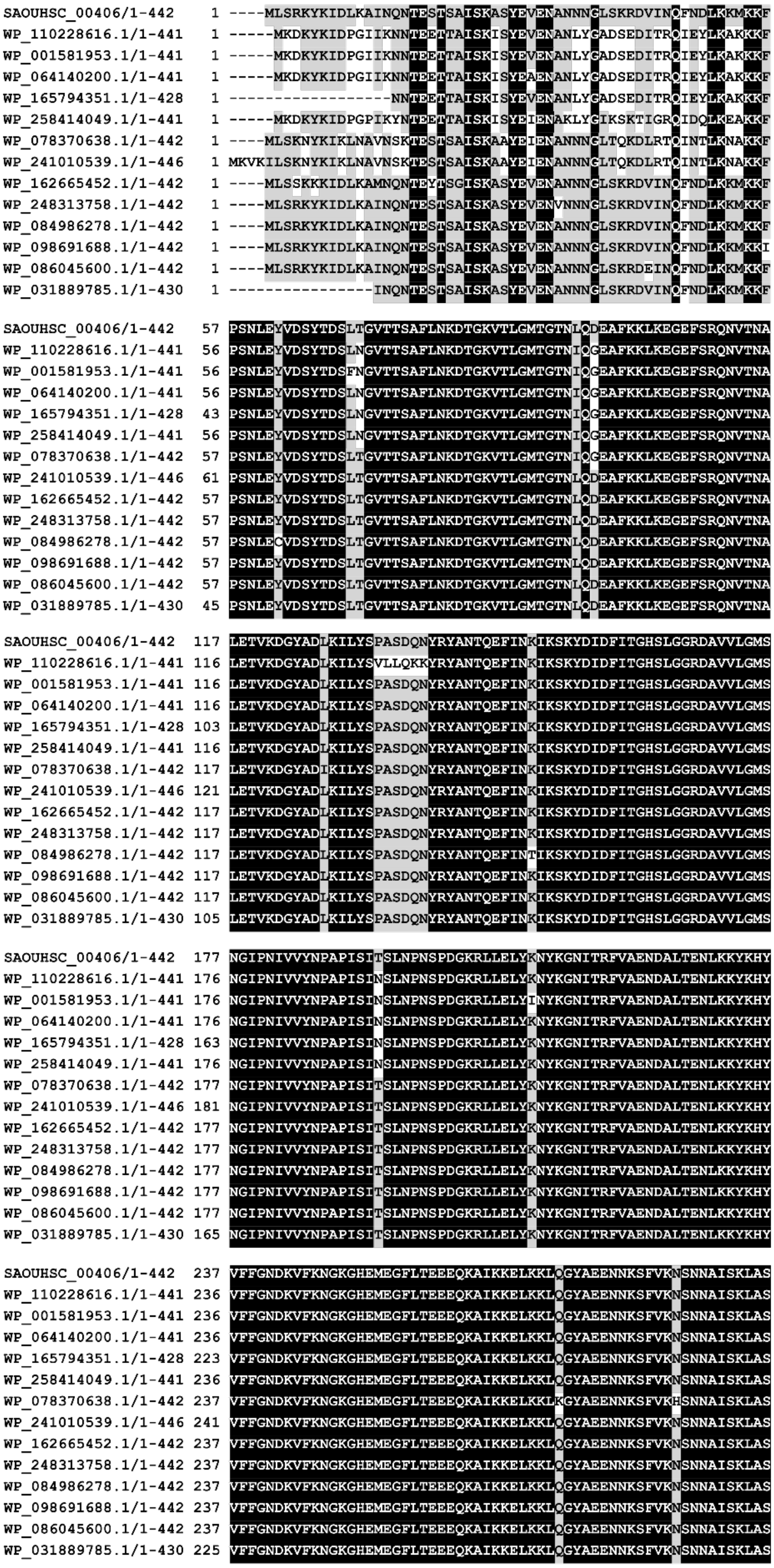

Supplementary Fig. 1. SAOUHSC_00406/TslA has a polymorphic N-terminal region. TslA homologues were identified by a blast search against the RefSeq database. The first 113 sequences were aligned using Muscle and visualised using Boxshade, with 13 representative sequences used to visualise the alignment of amino acids 1-296 of TslA.

a.

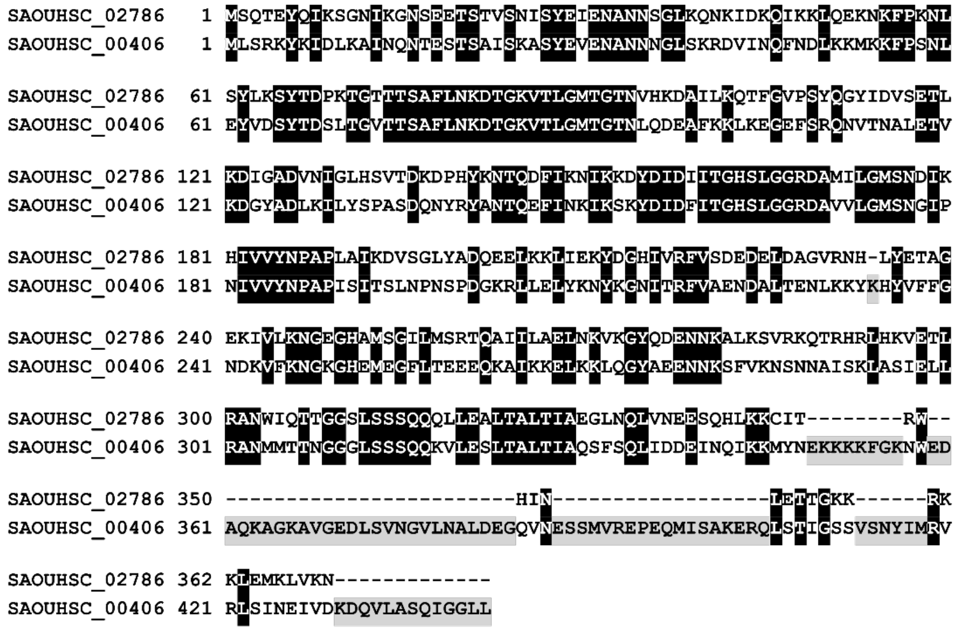

b.

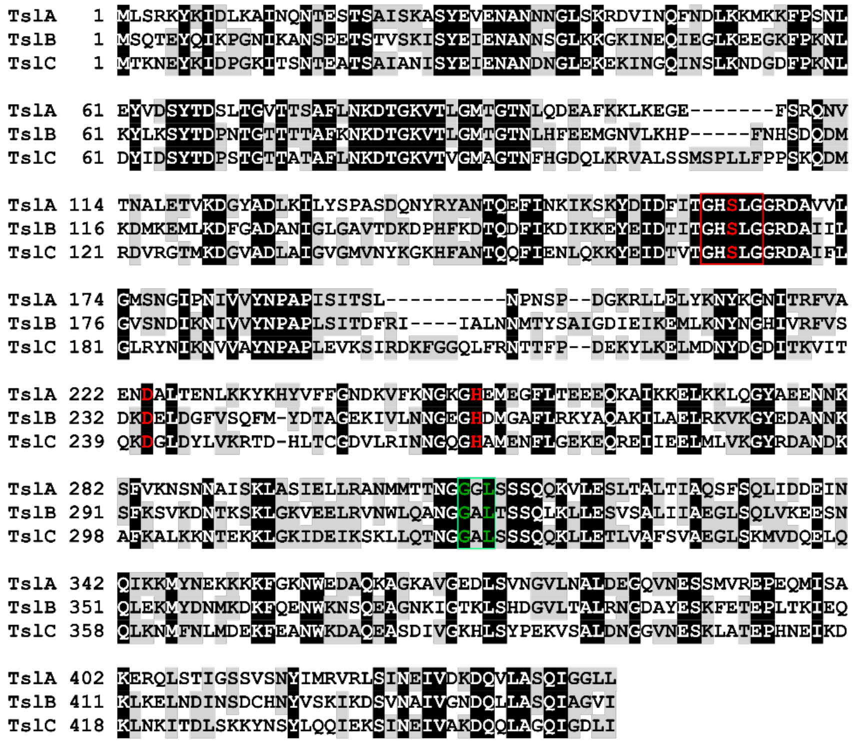

Supplementary Fig. 2. Two further homologues of TslA can be encoded in *S. aureus* genomes. a. Alignment of TslA with pseudogenous homologue, SAOUHSC_02786/TslB. b. Alignment of TslA with SAPIG_RS13365/TslB and CO08_0212/TslC. Residues indicated in red were mutated to alanine as part of this study. The G-X-S-X-G motif found in some type VI-secreted lipases is indicated by the red box. The putative G-X-L secretion motif is highlighted in green.

a.

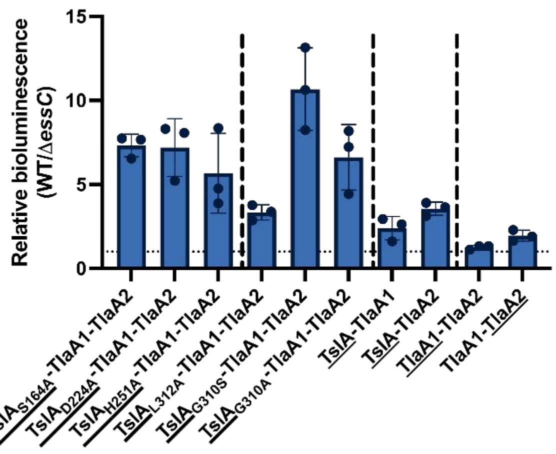

b.

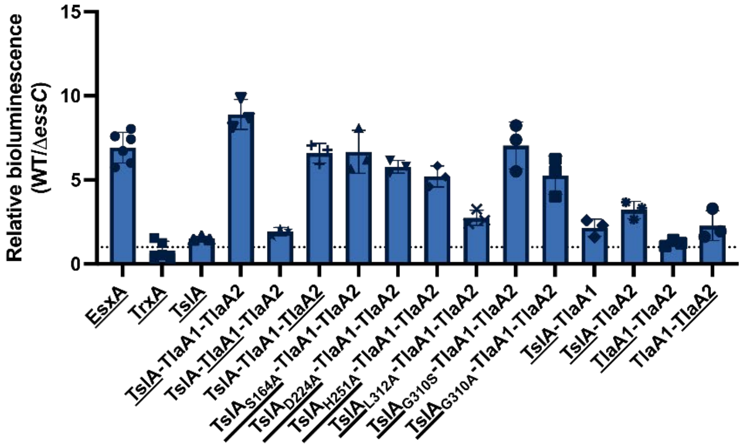

c.

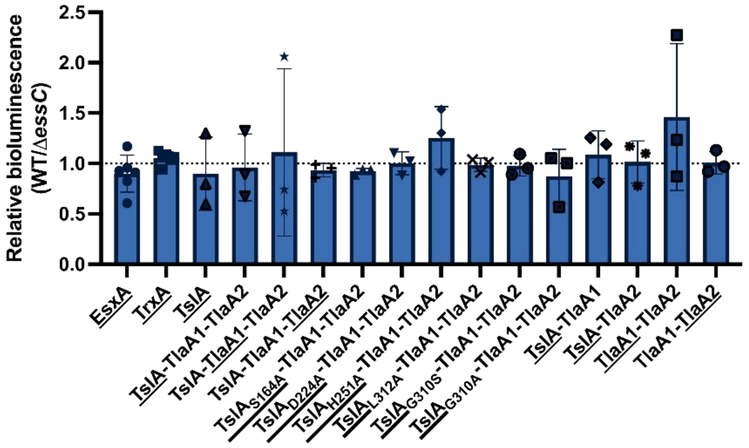

Supplementary Fig. 3. The split nanoluciferase assay indicates that TslA is detected in the culture supernatant when co-produced with TlaA1 and TlaA2. a. The relative luminescence present in whole cell samples producing the indicated proteins. The underline denotes the protein to which the pep86 fragment was fused. b. and c. Whole cell samples from the same experiment as Fig. 1e were processed into b. supernatant and c. cytoplasmic fractions as described in the methods. 11S fragment of nanoluciferase and furimazine were added, and luminescence readings taken over a 10 min time course, with peak readings used calculate relative bioluminescence for WT/*essC*. Experiments were performed in triplicate (n=3). Data presented as the mean ± SD. Two-way ANOVA was used to determine statistical

significance (n.s. p>0.05; **** p<0.0001). The experiment was performed a total of three to twelve times, dependent on the condition, all producing similar results.

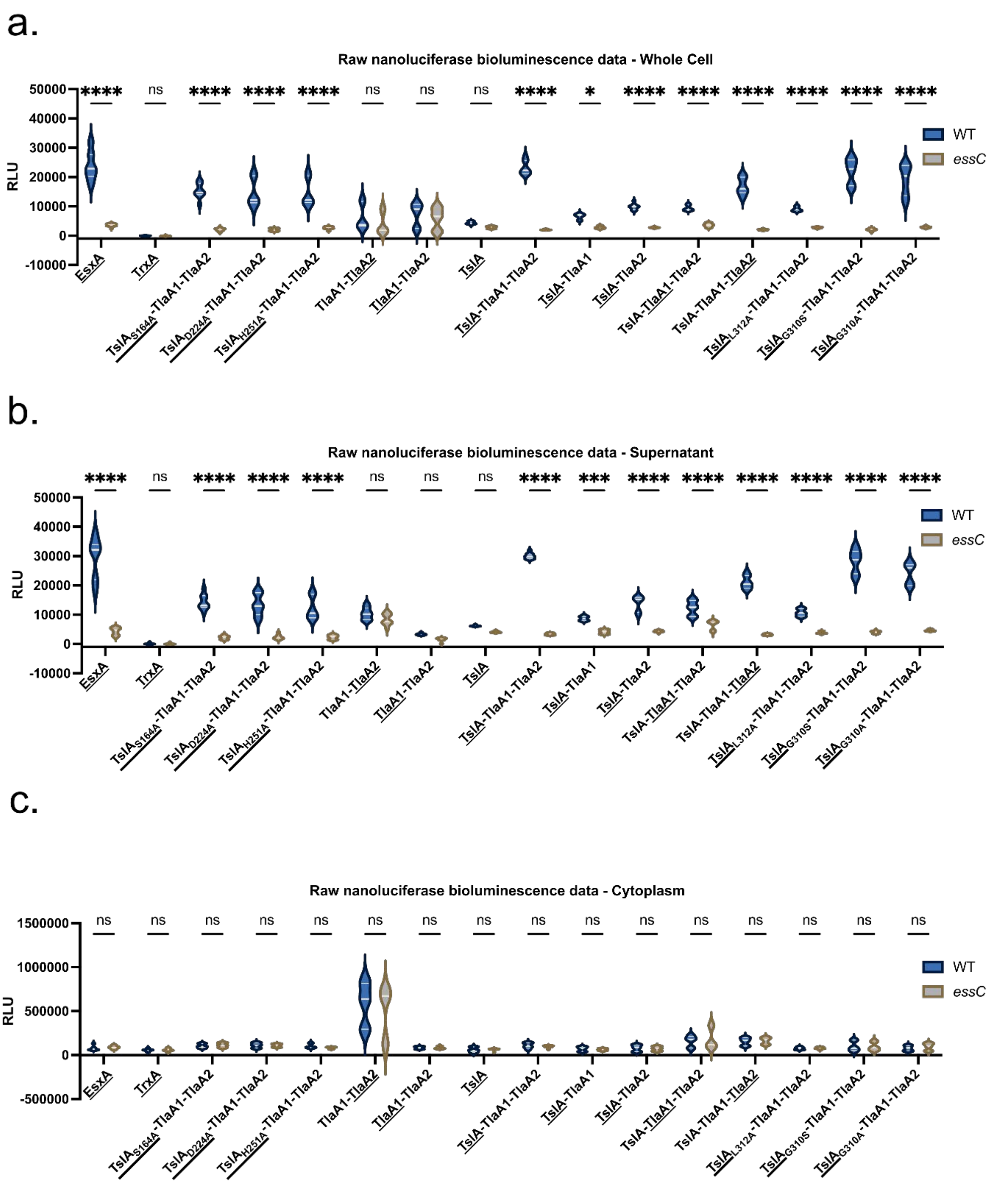

Supplementary Fig. 4. The raw data for the split nanoluciferase assays presented in Fig. 1e and Supplementary Fig 3. All normalised data for a. whole cell, b. supernatant and c. cytoplasmic fractions, used to represent the relative luciferase bioluminescence in previous figures, have been represented as violin plots. n=3 biological replicates. Error bars are ± SD. RLU – relative bioluminescence units.

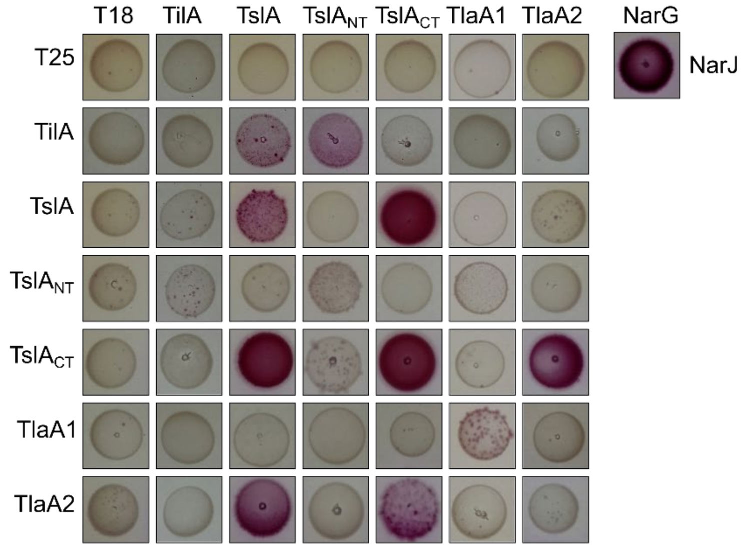

Supplementary Fig. 5. Bacterial two-hybrid analysis of the four proteins encoded at the *tslA* cluster. TilA, TslA (full length and separated N- and C-terminal domains), TlaA1 and TlaA2 were produced as fusions to the T18 and T25 fragments of *Bordetella pertussis* CyaA and pairwise interactions scored in the *E. coli cyaA* mutant strain BTH101. BTH101 producing these fusions was plated onto MacConkey agar supplemented with 1% maltose, and plates were photographed after 40 hours at 30°C. NarG-T18 and T25-NarJ were used as a positive control[15], and unfused T18 and T25 as a negative control. This experiment was repeated at least three times. Representative images are shown.

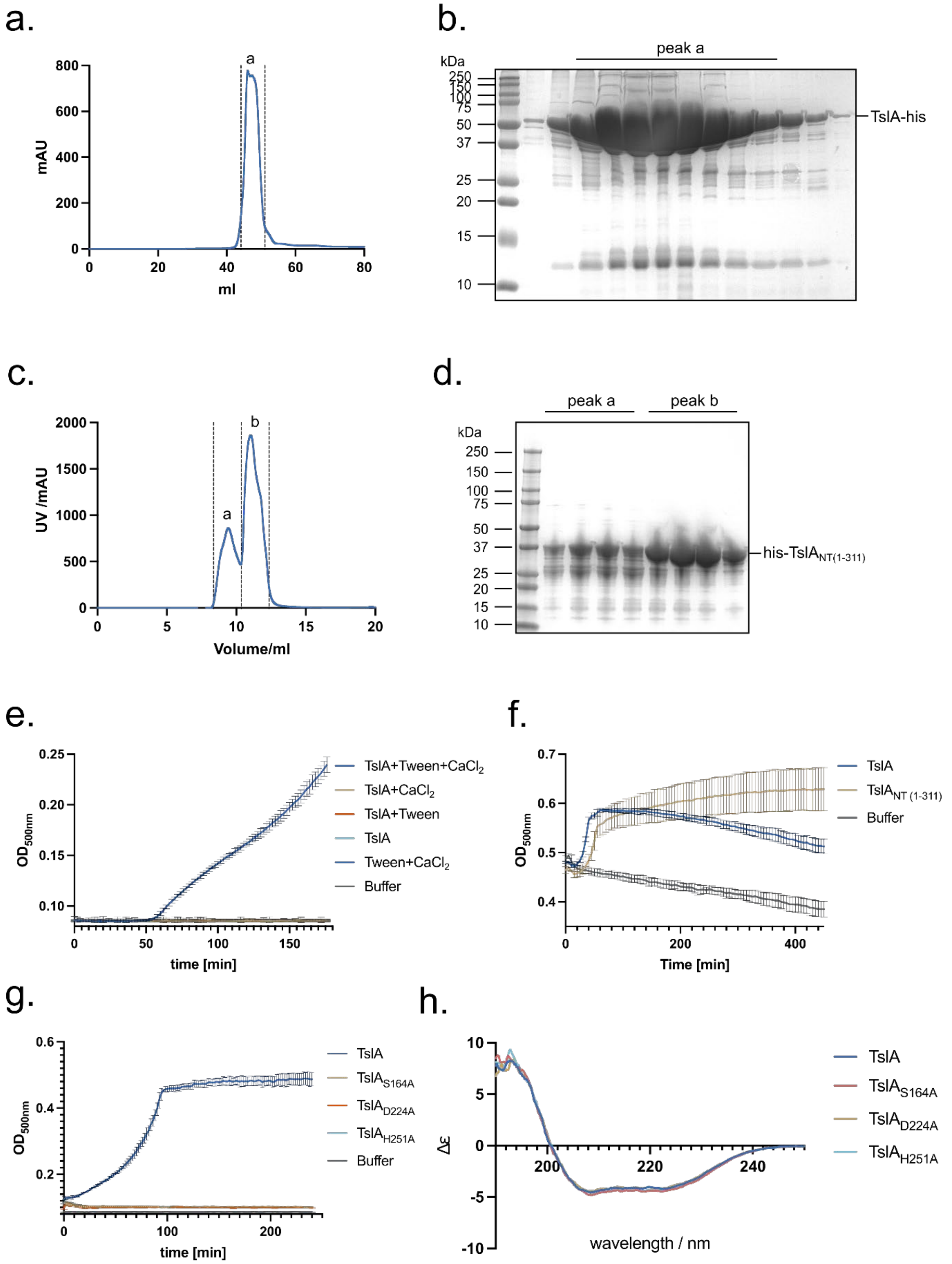

Supplementary Fig. 6. TslA and its isolated N-terminal domain have lipase activity against the model lipid substrate Tween 20. a. Size exclusion chromatogram of TslA-containing fractions that had been previously purified by Ni-affinity chromatography. (AU – absorbance units). b. SDS PAGE analysis of the peak fractions from a. The indicated fractions were pooled and used for activity assays. c. Size exclusion chromatogram of $TslA_{NT}$-containing fractions that had been previously purified by Ni-affinity chromatography. d. SDS

PAGE analysis of the two peak fractions from c. Fractions from peak b were pooled and used for activity assays. e-g. Tween 20 activity assays (carried out as described in the methods) with TslA, TslA amino acid substituted variants or $TslA_{NT}$ as indicated. Experiments were performed in technical and biological triplicates. Data is presented as the mean $\pm$ SD. The experiments were performed three times with similar results. h. Circular dichroism spectra for purified TslA and the S164A, D224A and H251A variants.

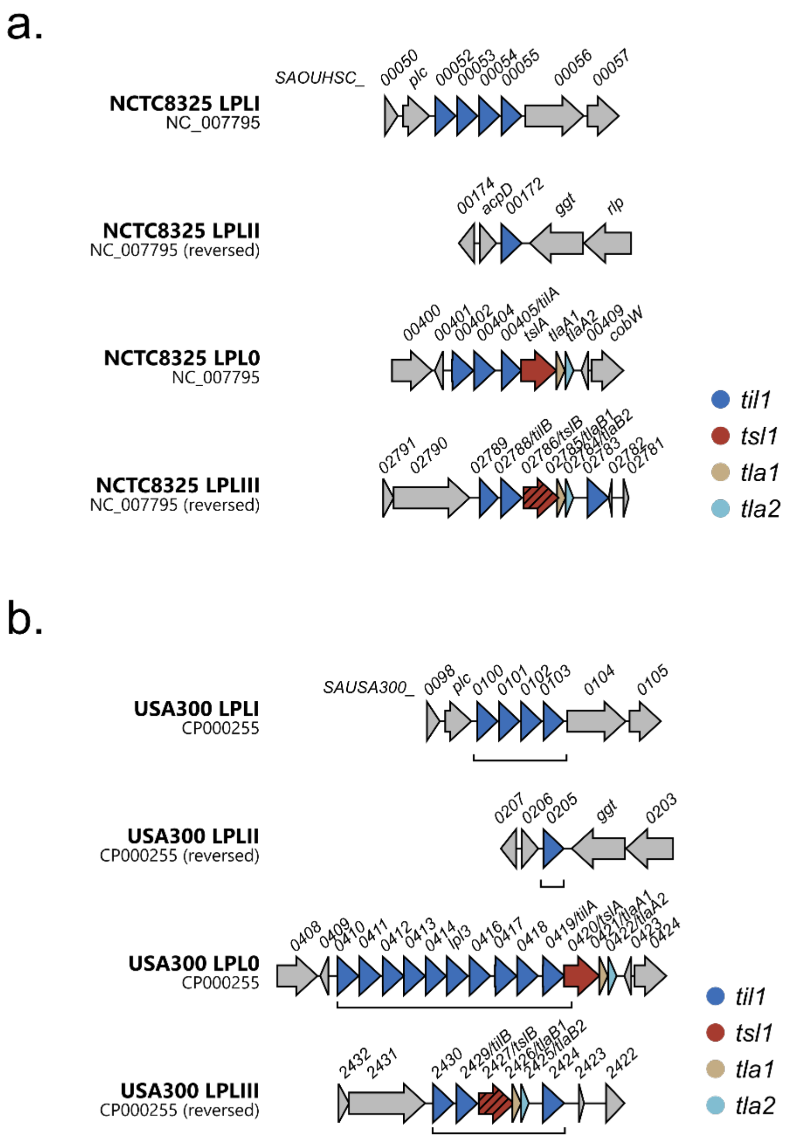

Supplementary Fig. 7. *S. aureus* strains encode three DUF576 tandem lipoprotein (Til1) islands and one orphan Til1. Til1 proteins are encoded at four loci in *S. aureus* genomes[18]. a. The loci shown here are from the NCTC8325 genome. The hatched shading indicates a probable pseudogene. Gene diagrams were visualised in Clinker[19]. b. The four Til1 loci from USA300 are depicted here. Black lines indicate genes deleted in the *til1*-deficient mutant.

a.

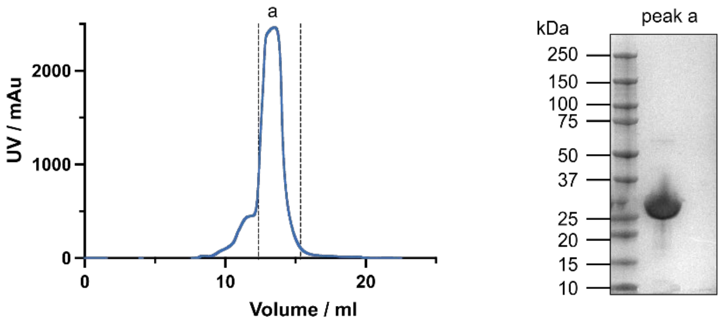

b.

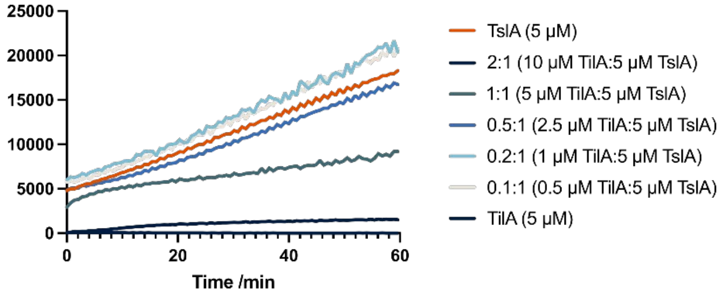

Supplementary Fig. 8. The TilA immunity protein inhibits TslA lipase activity. a, Size exclusion chromatogram of TilA-containing fractions that had been previously purified by Ni-affinity chromatography (left) and SDS PAGE analysis of the peak fraction (right). b, Purified TslA alone, TilA alone or TslA and TilA at the indicated molar ratios were incubated with the PLA_2 substrate PED6. Fluorescence released upon substrate hydrolysis was measured at 515 nm over the course of 1 h, and negative control values were subtracted from each condition.

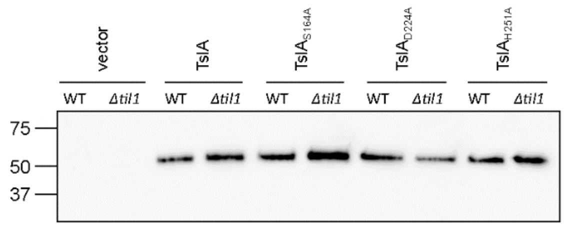

Supplementary Fig. 9. TslA and catalytically inactive variants are produced at similar levels in USA300 strains. USA300 and the isogenic *til1* mutant carrying pRAB11 (vector) or pRAB11 encoding TlaA1 and TlaA2 alongside the indicated variant of TslA were grown for 2 h post induction in TSB supplemented with 5 mM $CaCl_2$. The equivalent of 1 ml of culture of OD_{600} = 1 was withdrawn from each, pelleted and resuspended in PBS containing 1 mg ml^{-1} lysostaphin. Samples were incubated at 37°C for 30 min and boiled for 10 min in 2 X Laemmli buffer before analysis by SDS PAGE and Western blotting with anti-TslA antibodies.

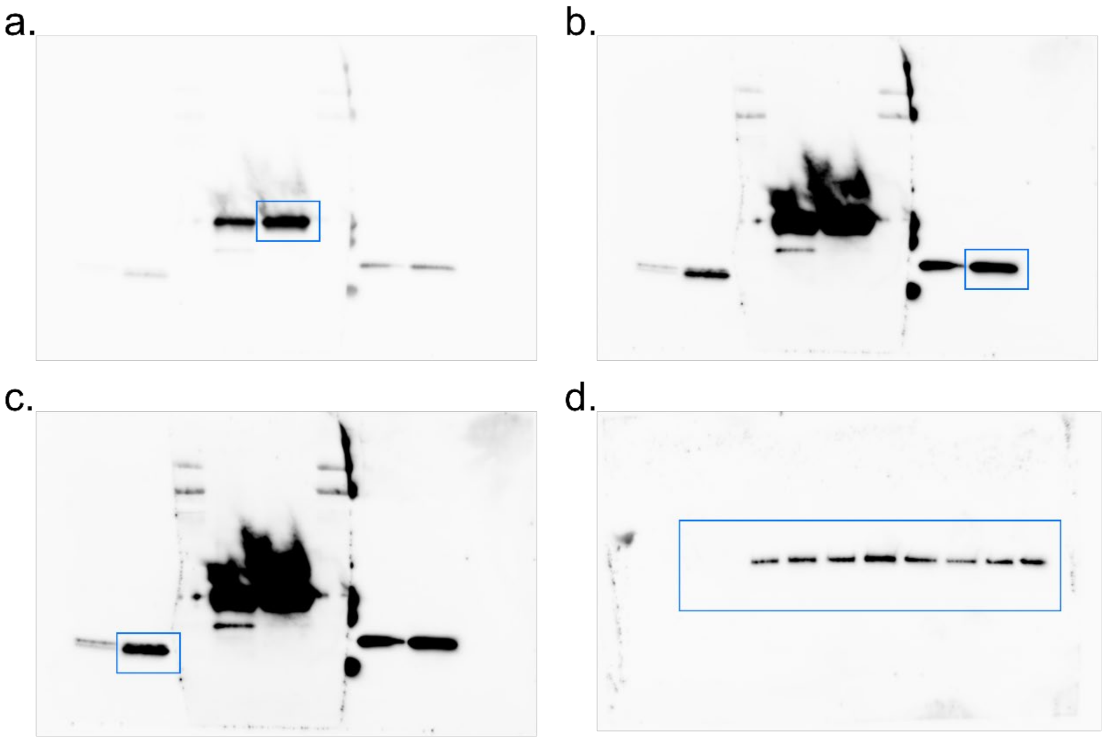

Supplementary Fig. 10. Uncropped Western blots. Purified fractions containing His-TslA$_{(277-442)}$, TlaA1-Strep and TlaA2-Myc following size exclusion chromatography were electrophoresed on a 4-20% SDS gel in triplicate, separated by a protein standard marker, and subsequently transferred to a nitrocellulose membrane, as shown in Fig. 2f. The membrane we cut at each of the two protein standards and each of membranes incubated with either TslA, Strep or Myc antibodies, as described in the methods. The membranes were placed together for imaging with a. 15 s, b. 4 min 30 s and c. 9 min exposure images used for TslA, Strep and Myc, respectively. d. Uncropped image of the TslA blot in Supplementary Fig. 9.

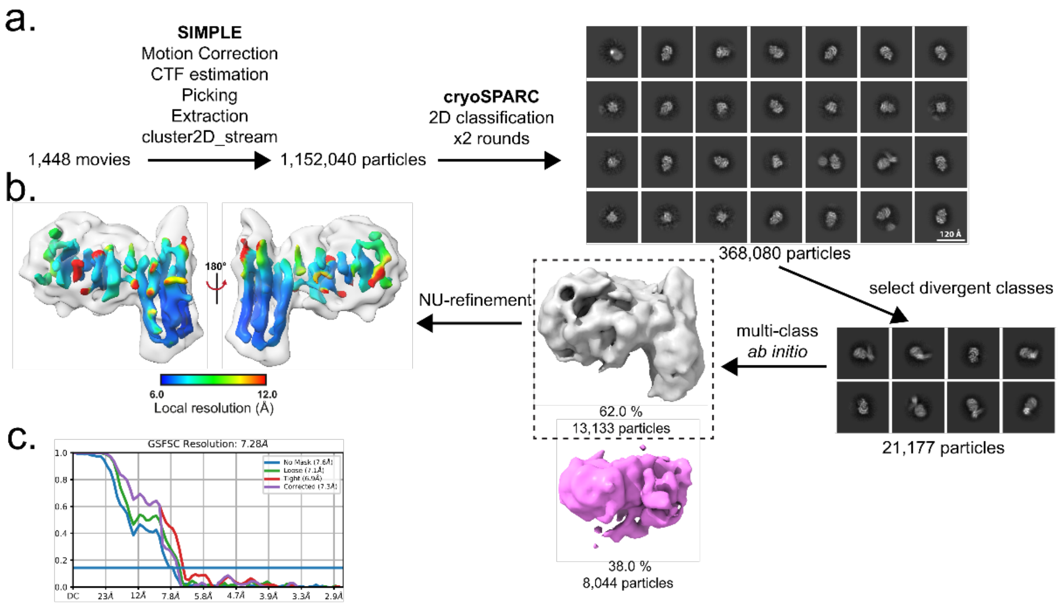

Supplementary Fig. 11. Cryo-EM processing workflow, showing local and global map quality. a, Image processing workflow. b, Local-resolution estimation of reconstructed map as determined within cryoSPARC. Map in transparent grey is shown at low contour level, whereas rainbow coloured map has been sharpened with a B-factor of -786 and displayed at higher contour level to show secondary structure elements. c, Gold-standard FSC curves used for global-resolution estimate as determined within cryoSPARC.

	Number of strains with full length toxin Present	Number of strains with toxin pseudogene present	Number of strains with toxin absent	Number of strains excluded due to lack of coverage
TslA	289	79	0	135
% of strains with TslA	78.5	21.5	0	
TslB	244	155	192	363
% of strains with TslB	41.3	26.2	32.5	
TslC	70	5	451	52
% of strains with TslC	13.3	1.0	85.7	

Supplementary Table 1. Analysis of Tsl1 distribution in *S. aureus* genomes. To assess the distribution of TslA, TslB and TslC across *S. aureus* strains, a conserved protein encoded at each of the LPL0 (SAOUHSC_00400), LPLIII (SAOUHSC_02790) and LPLI loci (SAOUHSC_00056), respectively, were searched against all *S. aureus* strains present in the RefSeq database. The output was filtered for >90% percent identity and coverage with the query sequence, to ensure the correct locus was selected, and the accession list submitted for flanking gene analysis using webFlags[1]. The toxin was recorded as present if an open reading frame was detected in the webFlags output, as pseudogenised if predicted to be a pseudogene by webFlags and absent if the toxin was not found between the conserved genes at either end of the LPL locus. If the contig was too short or if the conserved flanking genes for the locus were not present, the contig was excluded.

	USA300 positive control	USA300 negative control	USA300 TslA-TlaA1-TlaA2	USA300 Δ*til1* positive control	USA300 Δ*til1* negative control	USA300 Δ*til1* TslA-TlaA1-TlaA2
**Total cells**	1216	680	1538	1346	735	864
**Total cells with membrane damage**	560	8	3	905	1	179
**Percentage cells membrane damage**	46.05	1.18	0.20	67.24	0.14	20.72

Supplementary Table 2. Analysis of *S. aureus* USA300 cells stained by Sytox green when imaged using fluorescence microscopy. The percentage cells stained with Sytox green was calculated for each strain from the total cells analysed, as described in the methods. Data is represented in Fig. 5e.

Strain	Relevant genotype or description	Source or reference
E. coli		
JM110	*rpsL thr leu thi lacY galK galT ara tonA tsx dam dcm glnV44* Δ*(lac-proAB) e14-* [*F' traD36 proAB+ lacIq lacZ*Δ*M15*] *hsdR17(rK−mK+)*	Stratagene
TOP10	*F- mcrA (mrr-hsdRMS-mcrBC) 80lacZ M15 lacX74 recA1 ara 139 (ara-leu)7697 galU galK rpsL* (Str^r) *endA1 nupG*	Invitrogen
BL21(DE3)	*E. coli B: F$^-$, dcm, ompT, hsdS(rB-, mB-), gal,* λ(DE3)	Reference[2]
M15 [pREP4]	*F-, lac, ara, gal, mtl,* [Kan^r, *lacI*]	Qiagen
BTH101	F$^-$ *cya-99, araD139, galE15, galK16, rpsL1* (Str^r), *hsdR2, mcrA1, mcrB1*	Reference[3]
DH5a	φ80d Δ*(lacZ)M15 recA1 endA1 gyrA96 thi-1 hsdR17 (rk-mk+) supE44 relA1 deoR* Δ*(lacZYA-argF)*U169	Promega
S. aureus		
RN6390	NCTC8325 derivative, *rbsU, tcaR*, cured of φ11, φ12, φ13	Reference[4]
USA300 LAC	Wild type	Reference[5]
USA300 Δ*essC*	In-frame deletion of *essC* from USA300 LAC	This work
USA300 Δ*tslA*	In-frame deletion of *tslA* from USA300 LAC	This work
USA300 Δ*til1*	Also named *S. aureus* USA300 Δ*lpl*Δ*lpp3*Δ*lpp4*Δ*csa1*. In-frame deletions of all four LPL loci.	Reference[6]
USA300 Δ*til1* Δ*essC*	In-frame deletion of *essC* from USA300 Δ*til1*	This work
USA300 Δ*til1::tilA*	As USA300 *Δtil1*, with a copy of *tilA* introduced between *SAOUHSC_00037* and *SAOUHSC_00039* using pTH100_tilA.	This Work

Supplementary Table 3. Bacterial strains used in this work.

Plasmid	Relevant genotype or description	Source or reference
pIMAY	*E. coli/S. aureus* shuttle vector, temperature sensitive, cml^r	Reference[7]
pIMAY-essC	pIMAY carrying *essC* deletion allele	Reference[8]
pIMAY-Z	*E. coli/S. aureus* shuttle vector, temperature sensitive, cml^r	Reference[9]
pIMAY-Z-tslA	pIMAY carrying *tslA* deletion allele	This work
pTH100	Plasmid for markerless integration of GFP into *S. aureus*	Reference[10]
pTH100-tilA	As pTH100 but *gfp* replaced by *tilA,* including its native ribosome binding site (rbs)	This work
pRAB11	*E. coli/S. aureus* shuttle vector, inducible protein expression, *amp^r, cml^r*	Reference[11]
pRAB11-TslA	pRAB11 encoding *tslA*, preceded by *hla* rbs	This work
pRAB11-TslA-TlaA1-TlaA2	pRAB11 encoding *tslA-tlaA1-tlaA2*. The *hla* rbs precedes *tslA*	This work
pRAB11-pep86_TslA-TlaA1	As pRAB11 encoding *tslA-tlaA1-tlaA2*, with the *tlaA2* gene deleted. TslA fused to an N-terminal pep86 tag. The *hla* rbs precedes *tslA*. Synthesised by GenScript.	This work
pRAB11-pep86_TslA -TlaA2	As pRAB11 encoding *tslA-tlaA1-tlaA2*, with the *tlaA1* gene deleted. TslA fused to an N-terminal pep86 tag. The *hla* rbs precedes *tslA.* Synthesised by GenScript.	This work
pRAB11-TslA$_{S164A}$-TlaA1-TlaA2	As pRAB11-TslA-TlaA1-TlaA2 but encoding TslA S164A substitution	This work
pRAB11-TslA$_{D224A}$-TlaA1-TlaA2	As pRAB11-TslA-TlaA1-TlaA2 but encoding TslA D224A substitution	This work
pRAB11-TslA$_{H251A}$-TlaA1-TlaA2	As pRAB11-TslA-TlaA1-TlaA2 but encoding TslA H251A substitution.	This work
pRAB11-pep86_TslA$_{L312A}$-TlaA1-TlaA2	As pRAB11-TslA-TlaA1-TlaA2 but encoding TslA L312A substitution. TslA fused to an N-terminal pep86 tag. Synthesised by GenScript.	This work
pRAB11-pep86_TslA$_{G310A}$-TlaA1-TlaA2	As pRAB11-TslA-TlaA1-TlaA2 but encoding TslA G310A substitution. TslA fused to an N-terminal pep86 tag. Synthesised by GenScript.	This work
pRAB11-pep86_TslA$_{G310S}$-TlaA1-TlaA2	As pRAB11-TslA-TlaA1-TlaA2 but encoding TslA G310S substitution. TslA fused to an N-terminal pep86 tag. Synthesised by GenScript.	This work
pRAB11-pep86_EsxA	pRAB11 producing EsxA fused to an N-terminal pep86 tag. The *hla* rbs precedes *esxA*	This Work
pRAB11-pep86_TrxA	pRAB11 producing TrxA fused to an N-terminal pep86 tag. The *hla* rbs precedes *trxA*	Reference[12]
pRAB11-pep86_TslA	pRAB11 producing TslA fused to an N-terminal pep86 tag. The *hla* rbs precedes *tslA*. Synthesised by GenScript.	This Work
pRAB11-pep86_TslA-TlaA1-TlaA2	pRAB11 encoding *tslA-tlaA1-tlaA2*, producing TslA fused to an N-terminal pep86 tag. The *hla* rbs precedes *tslA*	This Work
pRAB11-TslA-TlaA1-pep86_TlaA2	pRAB11 encoding *tslA-tlaA1-tlaA2*, producing TlaA2 fused to an N-terminal pep86 tag. The *hla* ribosome binding site precedes both *tslA* and *tlaA2*	This Work
pRAB11-TslA-TlaA1_pep86-TlaA2	pRAB11 encoding *tslA-tlaA1-tlaA2*, producing TlaA1 fused to an C-terminal pep86 tag. The *hla* rbs precedes *tslA*. Synthesised by GenScript.	This work
pRAB11-pep86_TslA$_{S164A}$-TlaA1-TlaA2	As pRAB11-TslA-TlaA1-TlaA2 but encoding TslA S164A substitution. TslA fused to an N-terminal pep86 tag.	This work
pRAB11-pep86_TslA$_{D224A}$-TlaA1-TlaA2	As pRAB11-TslA-TlaA1-TlaA2 but encoding TslA D224A substitution. TslA fused to an N-terminal pep86 tag.	This work
pRAB11-pep86_TslA$_{H251A}$-TlaA1-TlaA2	As pRAB11-TslA-TlaA1-TlaA2 but encoding TslA H251A substitution. TslA fused to an N-terminal pep86 tag.	This work
pRAB11- TlaA1-pep86_TlaA2	pRAB11 encoding *tlaA1-tlaA2*, producing TlaA2 fused to an N-terminal pep86 tag. The *hla* ribosome binding site precedes both *tlaA1* and *tlaA2*	This Work
pRAB11-TlaA1_pep86-TlaA2	pRAB11 encoding *tslA-tlaA1-tlaA2*, producing TlaA1 fused to an C-terminal pep86 tag. The *hla* rbs precedes *tlaA1.*	This work
pBAD-$_{6H}$11S	Expression vector for purification of 11S	Reference[13]

pQE70	Vector for regulatable protein overproduction in *E. coli* (T5 promoter). amp^r	Qiagen
pQE70-TslA-His	pQE70 with *tslA* cloned in frame in the multiple cloning site to produce a C-terminal His(6)-tag fusion.	This Work
pQE70-TslA$_{S164A}$-His	As pQE70-TslA-His but encoding TslA S164A substitution	This Work
pQE70-TslA$_{D224A}$-His	As pQE70-TslA-His but encoding TslA D224A substitution	This Work
pQE70-TslA$_{H251A}$-His	As pQE70-TslA-His but encoding TslA H521A substitution	This Work
pREP4	*lacI* kan^r	Qiagen
pLysS	Encodes T7 lysozyme cml^r	Promega
pET15bTEV	Overexpression plasmid, T7 promoter. Adds (His)6-tag and a TEV site to N-terminus of protein	Reference[14]
pET15bTEV-TslA$_{CT}$-TlaA1-Strep-TlaA2-Myc	pET15bTEV producing TslA lacking the first 276 amino acids as an N-terminal His(6)-fusion, alongside TlaA1 with a C-terminal strep tag and TlaA2 with a C-terminal Myc tag	This work
pET15bTEV-His-TilA-TslA-TlaA1-Strep-TlaA2-Myc	pET15bTEV producing TilA lacking the first 39 amino acids as an N-terminal His(6)-tag fusion, alongside full length TslA, TlaA1 with a C-terminal strep tag and TlaA2 with a C-terminal Myc tag	This work
pET15bTEV-His-TilA	pET15bTEV producing TilA lacking the first 40 amino acids as an N-terminal His(6)-tag fusion	This Work
pET15bTEV-His-TslA$_{NT(1-311)}$	pET15bTEV producing the N-terminal domain of TslA (aa 1 – 311) with an N-terminal His(6)-tag	This Work
pUT18	Vector encoding T18 fragment of *B. pertussis* CyaA; amp^r	Reference[15]
pUT18-NarG	aa 1-42 of *E. coli* NarG fused to the N-terminus of T18 CyaA	Reference[16]
pUT18-TilA	Mature region of TilA (i.e. lacking aa 1-24) fused to the N-terminus of T18 CyaA	This Work
pUT18-TslA	TslA fused to the N-terminus of T18 CyaA	This Work
pUT18-TslA$_{NT}$	N-terminal domain of TslA (aa 1-260) fused to the N-terminus of T18 CyaA	This Work
pUT18-TslA$_{CT}$	C-terminal domain of TslA (aa 239-442) fused to the N-terminus of T18 CyaA	This Work
pUT18-TlaA1	TlaA1 fused to the N-terminus of T18 CyaA	This Work
pUT18-TlaA2	TlaA2 fused to the N-terminus of T18 CyaA	This Work
pT25	Vector encoding T25 fragment of *B. pertussis* CyaA cml^r	Reference[17]
pT25-NarJ	*E. coli* NarJ fused to the C-terminus of T25 CyaA	Reference[16]
pT25-TilA	Mature region of TilA (i.e. lacking aa 1-25) fused to the C-terminus of T25 CyaA	This Work
pT25-TslA	TslA fused to the C-terminus of T25 CyaA	This Work
pT25-TslA$_{NT}$	N-terminal domain of TslA (aa 2-260) fused to the C-terminus of T25 CyaA	This Work
pT25-TslA$_{CT}$	C-terminal domain of TslA (aa 239-442) fused to the C-terminus of T25 CyaA	This Work
pT25-TlaA1	TlaA1 fused to the C-terminus of T25 CyaA	This Work
pT25-TlaA2	TlaA2 fused to the C-terminus of T25 CyaA	This Work

Supplementary Table 4. Plasmids used in this work.

Protein	Expression time	Sample application AC	Buffer	Column
TslA (wildtype and point substituted variants)	4 h	2.5 ml min^{-1} supplemented with 20 mM imidazole	Buffer A: 50 mM HEPES pH 7.5 300 mM NaCl Eluted with a gradient of 0 – 500 mM imidazole in Buffer A	5 ml HisTrap FF
			Buffer B: 20 mM HEPES pH 7.5150 mM NaCl	Superdex® 75 pg 16/600
TslA$_{CT}$-TlaA1-TlaA2	4 h	2 ml min^{-1} supplemented with 50 mM imidazole	Buffer A: 50 mM HEPES pH 8, 150 mM NaCl Eluted with a gradient of 50 – 500 mM imidazole in Buffer A	5 ml HisTrap FF
			Buffer A: 50 mM HEPES pH 8, 150 mM NaCl Step elution with 5 mM *d*-desthiobiotin in Buffer A	1ml StrepTrap, FF
			Buffer B: 50 mM HEPES pH 8, 150 mM NaCl	Superdex® 75 pg 16/600
TilA-TslA-TlaA1-TlaA2	2.5 h	0.5 ml min^{-1} supplemented with 20 mM imidazole	Buffer A: 50 mM HEPES pH 7.5, 300 mM NaCl Eluted with a gradient of 0 – 500 mM imidazole in Buffer A	1 ml HisTrap, FF
			Buffer A: 50 mM HEPES pH 7.5, 300 mM NaCl Step elution with 5 mM *d*-desthiobiotin in Buffer A	1ml StrepTrap, FF
			Buffer B: 20 mM HEPES pH 7.5, 150 mM NaCl,	Superdex® 200 10/300 GL
TilA	2 h	2.5 ml min^{-1} supplemented with 20 mM imidazole	Buffer A: 50 mM HEPES pH 7.5, 300 mM NaCl Eluted with a gradient of 0 – 500 mM imidazole in Buffer A	5 ml HisTrap, FF
			Buffer B: 20 mM HEPES pH 7.5, 150 mM NaCl	Superdex® 75 pg 10/300
11S-His	4 h	2.5 ml min^{-1} supplemented with 50 mM imidazole	Buffer A: 20 mM Tris HCl pH 7.5, 50 mM KCl, 10% glycerol (v/v) Eluted with a gradient of 50 – 550 mM imidazole in Buffer A	5 ml HisTrap FF
			Buffer B: 50 mM HEPES pH 8, 150 mM NaCl	Superdex® 75 pg 16/600

Supplementary Table 5. Protein expression and purification conditions used in this work.

Supplementary References

1. Saha, C.K., Sanches Pires, R., Brolin, H., Delannoy, M. & Atkinson, G.C. FlaGs and webFlaGs: discovering novel biology through the analysis of gene neighbourhood conservation. *Bioinformatics* **37**, 1312-1314 (2021).

2. Studier, F.W. & Moffatt, B.A. Use of bacteriophage T7 RNA polymerase to direct selective high-level expression of cloned genes. *J Mol Biol* **189**, 113-130 (1986).

3. Karimova, G., Ullmann, A. & Ladant, D. A bacterial two-hybrid system that exploits a cAMP signaling cascade in *Escherichia coli*. *Methods Enzymol* **328**, 59-73 (2000).

4. Novick, R.P. et al. Synthesis of *staphylococcal* virulence factors is controlled by a regulatory RNA molecule. *EMBO J* **12**, 3967-3975 (1993).

5. Kazakova, S.V. et al. A clone of methicillin-resistant *Staphylococcus aureus* among professional football players. *N Engl J Med* **352**, 468-475 (2005).

6. Belikova, D., Jochim, A., Power, J., Holden, M.T.G. & Heilbronner, S. "Gene accordions" cause genotypic and phenotypic heterogeneity in clonal populations of *Staphylococcus aureus*. *Nat Commun* **11**, 3526 (2020).

7. Monk, I.R., Shah, I.M., Xu, M., Tan, M.W. & Foster, T.J. Transforming the untransformable: application of direct transformation to manipulate genetically *Staphylococcus aureus* and *Staphylococcus epidermidis*. *mBio* **3**, e00277-11 (2012).

8. Kneuper, H. et al. Heterogeneity in *ess* transcriptional organization and variable contribution of the Ess/Type VII protein secretion system to virulence across closely related *Staphylocccus aureus* strains. *Mol Microbiol* **93**, 928-943 (2014).

9. Monk, I.R. & Stinear, T.P. From cloning to mutant in 5 days: rapid allelic exchange in *Staphylococcus aureus*. *Access Microbiol* **3**, 000193 (2021).

10. de Jong, N.W., van der Horst, T., van Strijp, J.A. & Nijland, R. Fluorescent reporters for markerless genomic integration in *Staphylococcus aureus*. *Sci Rep* **7**, 43889 (2017).

11. Helle, L. et al. Vectors for improved Tet repressor-dependent gradual gene induction or silencing in *Staphylococcus aureus*. *Microbiology* **157**, 3314-3323 (2011).

12. Yang, Y., Alcock, F., Kneuper, H. & Palmer, T. A high throughput assay to measure Type VII secretion in *Staphylococcus aureus*. *Biorxiv* https://doi.org/10.1101/2023.06.03.543475 (2023).

13. Pereira, G.C. et al. A high-resolution luminescent assay for rapid and continuous monitoring of protein translocation across biological membranes. *J Mol Biol* **431**, 1689-1699 (2019).

14. Casabona, M.G. et al. Functional analysis of the EsaB component of the *Staphylococcus aureus* Type VII secretion system. *Microbiology* **163**, 1851-1863 (2017).

15. Karimova, G., Ullmann, A. & Ladant, D. Protein-protein interaction between *Bacillus stearothermophilus* tyrosyl-tRNA synthetase subdomains revealed by a bacterial two-hybrid system. *J Mol Microbiol Biotechnol* **3**, 73-82 (2001).

16. Ize, B. et al. Remnant signal peptides on non-exported enzymes: implications for the evolution of prokaryotic respiratory chains. *Microbiology* **155**, 3992-4004 (2009).

17. Karimova, G., Pidoux, J., Ullmann, A. & Ladant, D. A bacterial two-hybrid system based on a reconstituted signal transduction pathway. *Proc Natl Acad Sci U S A* **95**, 5752-5756 (1998).

18. Tsuru, T. & Kobayashi, I. Multiple genome comparison within a bacterial species reveals a unit of evolution spanning two adjacent genes in a tandem paralog cluster. *Mol Biol Evol* **25**, 2457-2473 (2008).

19. Gilchrist, C.L.M. & Chooi, Y.H. Clinker & clustermap.js: Automatic generation of gene cluster comparison figures. *Bioinformatics* **37**, 2473-2475 (2021).
