# Supplementary material for: A type VII-secreted lipase toxin with reverse domain arrangement
Source: Nat Commun. 2023 Dec 19;14:8438. doi: 10.1038/s41467-023-44221-y (PMC10730906; doi:10.1038/s41467-023-44221-y)
Supplement: Supplementary file 3 — Description of Additional Supplementary Files [file 41467_2023_44221_MOESM3_ESM.pdf]

Description of Additional Supplementary Files

Supplementary Data 1: Oligonucleotides used in this work. Underlined sequences indicate restriction endonuclease sites.
