# Supplementary material for: A type VII-secreted lipase toxin with reverse domain arrangement
Source: Nat Commun. 2023 Dec 19;14:8438. doi: 10.1038/s41467-023-44221-y (PMC10730906; doi:10.1038/s41467-023-44221-y)
Supplement: Supplementary file 5 — Reporting Summary [file 41467_2023_44221_MOESM5_ESM.pdf]

nature portfolio

Corresponding author(s): Tracy Palmer

Last updated by author(s): Nov 28, 2023

Reporting Summary

Nature Portfolio wishes to improve the reproducibility of the work that we publish. This form provides structure for consistency and transparency in reporting. For further information on Nature Portfolio policies, see our Editorial Policies and the Editorial Policy Checklist.

Statistics

For all statistical analyses, confirm that the following items are present in the figure legend, table legend, main text, or Methods section.

n/a	Confirmed	
☐	☒	The exact sample size (*n*) for each experimental group/condition, given as a discrete number and unit of measurement
☐	☒	A statement on whether measurements were taken from distinct samples or whether the same sample was measured repeatedly
☐	☒	The statistical test(s) used AND whether they are one- or two-sided<br>*Only common tests should be described solely by name; describe more complex techniques in the Methods section.*
☒	☐	A description of all covariates tested
☒	☐	A description of any assumptions or corrections, such as tests of normality and adjustment for multiple comparisons
☐	☒	A full description of the statistical parameters including central tendency (e.g. means) or other basic estimates (e.g. regression coefficient) AND variation (e.g. standard deviation) or associated estimates of uncertainty (e.g. confidence intervals)
☐	☒	For null hypothesis testing, the test statistic (e.g. *F*, *t*, *r*) with confidence intervals, effect sizes, degrees of freedom and *P* value noted<br>*Give P values as exact values whenever suitable.*
☒	☐	For Bayesian analysis, information on the choice of priors and Markov chain Monte Carlo settings
☒	☐	For hierarchical and complex designs, identification of the appropriate level for tests and full reporting of outcomes
☒	☐	Estimates of effect sizes (e.g. Cohen's *d*, Pearson's *r*), indicating how they were calculated

Our web collection on statistics for biologists contains articles on many of the points above.

Software and code

Policy information about availability of computer code

Data collection	*No software was used*
Data analysis	*Graphpad Prism v9.5.1 was used to graph results and perform statistical analysis. PyMOL V2.1.076 and Chimera X V1.464 were used for the analysis of protein structural models. Protein alignments were carried out using MUSCLE v3.8.155177.*

For manuscripts utilizing custom algorithms or software that are central to the research but not yet described in published literature, software must be made available to editors and reviewers. We strongly encourage code deposition in a community repository (e.g. GitHub). See the Nature Portfolio guidelines for submitting code & software for further information.

Data

Policy information about availability of data

All manuscripts must include a data availability statement. This statement should provide the following information, where applicable:

- Accession codes, unique identifiers, or web links for publicly available datasets
- A description of any restrictions on data availability
- For clinical datasets or third party data, please ensure that the statement adheres to our policy

Data generated or analysed in this study are available within the article and its supplementary materials. Source data are provided with this paper with the exception of the microscopy images which are available at FigShare (https://doi.org/10.6084/m9.figshare.24717864.v1). Databases used are Alphafold (https://alphafold.ebi.ac.uk/), Refseq (https://www.ncbi.nlm.nih.gov/refseq/) and Lipid maps (https://www.lipidmaps.org).

Research involving human participants, their data, or biological material

Policy information about studies with human participants or human data. See also policy information about sex, gender (identity/presentation), and sexual orientation and race, ethnicity and racism.

Reporting on sex and gender	N/A
Reporting on race, ethnicity, or other socially relevant groupings	N/A
Population characteristics	N/A
Recruitment	N/A
Ethics oversight	N/A

Note that full information on the approval of the study protocol must also be provided in the manuscript.

Field-specific reporting

Please select the one below that is the best fit for your research. If you are not sure, read the appropriate sections before making your selection.

☒ Life sciences ☐ Behavioural & social sciences ☐ Ecological, evolutionary & environmental sciences

For a reference copy of the document with all sections, see nature.com/documents/nr-reporting-summary-flat.pdf

Life sciences study design

All studies must disclose on these points even when the disclosure is negative.

Sample size	In vivo experiments were performed in line with our ethical allowance (IMIT01/20G). Numbers were determined using Biometric analysis of means and variances of Pilot-experiments, binding us to the use of 5 mice per group with two abscesses per mouse.
Data exclusions	Two individual values (out of a total of 18) were excluded from the relative luminescence values present for TrxA in Supplementary Fig 3c. This is because the luminescence values for TrxA in the WT and essC mutant were both so low that on these two occasions they greatly skewed the data. These values were, however, included in the raw data presented in Supplementary Fig. 4c.
Replication	All experiments were repeated a minimum of three times, all attempts at replication were successful. For growth curves and the nanoluciferase experiments, three biological replicates were performed, each with three technical replicates. For lipase assays, three technical replicates were performed. Individual abcesses in mice represent biological replicates of infection.
Randomization	Allocation of mice to groups infected with different strains was random.
Blinding	Blinding for in vivo experiments was performed as the experimenter was not informed about the strain the individual mice were infected with.

Reporting for specific materials, systems and methods

We require information from authors about some types of materials, experimental systems and methods used in many studies. Here, indicate whether each material, system or method listed is relevant to your study. If you are not sure if a list item applies to your research, read the appropriate section before selecting a response.

Materials & experimental systems

n/a	Involved in the study	
☐	☒	Antibodies
☒	☐	Eukaryotic cell lines
☒	☐	Palaeontology and archaeology
☐	☒	Animals and other organisms
☒	☐	Clinical data
☒	☐	Dual use research of concern
☒	☐	Plants

Methods

n/a	Involved in the study	
☒	☐	ChIP-seq
☒	☐	Flow cytometry
☒	☐	MRI-based neuroimaging

Antibodies

Antibodies used	Rabbit polyclonal anti-TslA (Davids Biotechnologie - raised using purified TslA-His supplied by the authors)<br>Mouse Anti-Myc (Abcam: ab23)<br>Mouse Anti-Strep (Qiagen: 34850)<br>Goat anti-rabbit IgG HRP conjugate (BioRad: 1721019)<br>Goat anti-mouse IgG HRP conjugate (BioRad: 1721011)
Validation	Anti-TslA antibody was validated against a strain lacking Tsl proteins.

Animals and other research organisms

Policy information about studies involving animals; ARRIVE guidelines recommended for reporting animal research, and Sex and Gender in Research

Laboratory animals	C57BL/6JRccHsd mice, 6 weeks old
Wild animals	No wild animals were used in the study
Reporting on sex	Female. Previous experiments using this model have utilised only female mice. For comparison with existing datasets female mice were used
Field-collected samples	No field collected samples were used in the study
Ethics oversight	Animal models were approved by the "Regierungspräsidium Tübingen" under the application number IMIT01/20G.

Note that full information on the approval of the study protocol must also be provided in the manuscript.

Plants

Seed stocks	*N/A*
Novel plant genotypes	*N/A*
Authentication	*N/A*
