# Supplementary material for: A type VII-secreted lipase toxin with reverse domain arrangement
Source: Nat Commun. 2023 Dec 19;14:8438. doi: 10.1038/s41467-023-44221-y (PMC10730906; doi:10.1038/s41467-023-44221-y)
Supplement: Supplementary file 6 — Source Data [file 41467_2023_44221_MOESM6_ESM.zip › Tsl1 distribution raw/lpl0 2/FlaGs_output/results_TreeOrder_output.pdf]

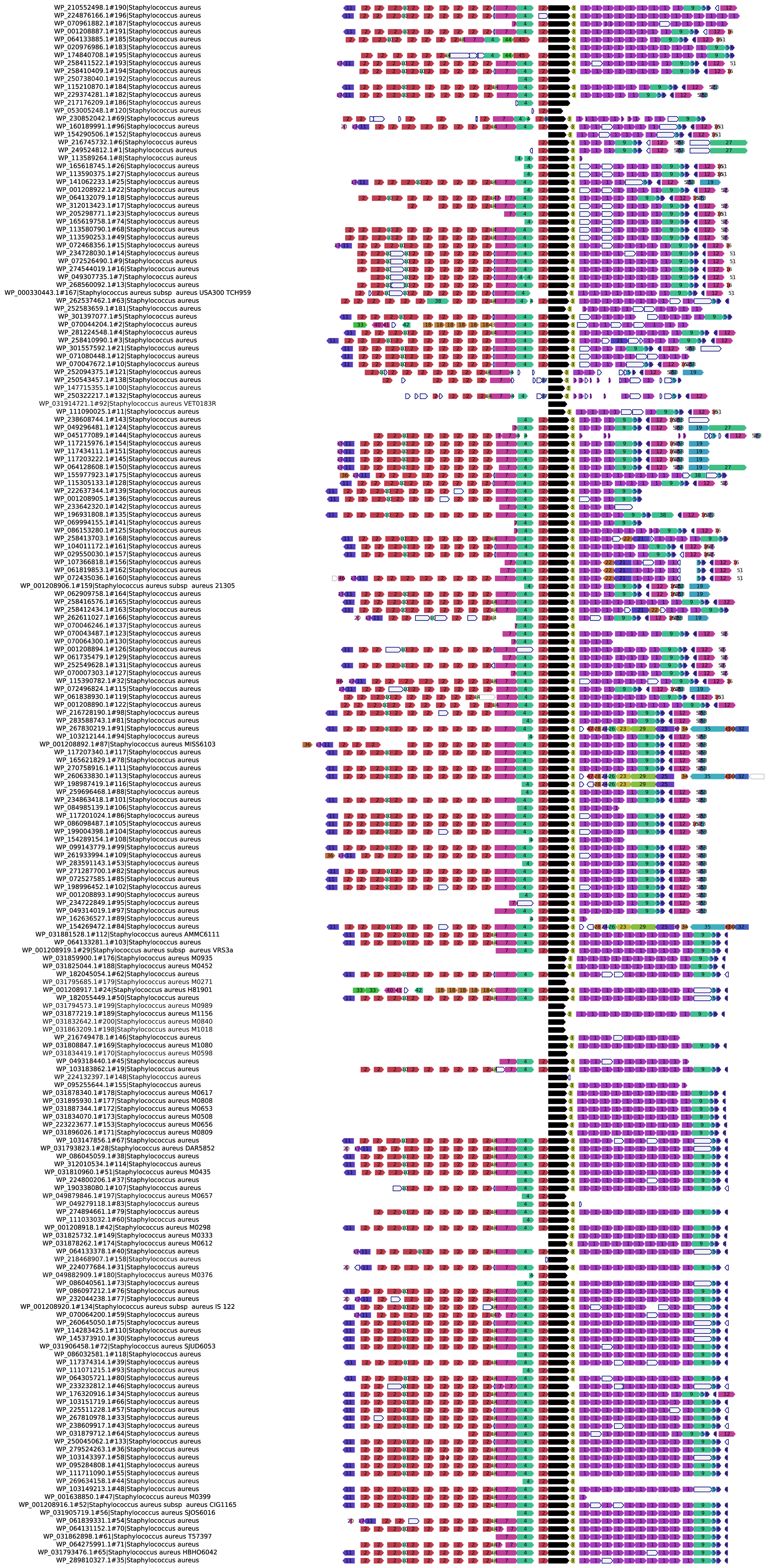
WP_210552498.1#190|Staphylococcus aureus
WP_224876166.1#196|Staphylococcus aureus
WP_070961882.1#187|Staphylococcus aureus
WP_001208887.1#191|Staphylococcus aureus
WP_064133885.1#185|Staphylococcus aureus
WP_020976986.1#183|Staphylococcus aureus
WP_174840708.1#195|Staphylococcus aureus
WP_258411522.1#193|Staphylococcus aureus
WP_258410409.1#194|Staphylococcus aureus
WP_250738040.1#192|Staphylococcus aureus
WP_115210870.1#184|Staphylococcus aureus
WP_229374281.1#182|Staphylococcus aureus
WP_217176209.1#186|Staphylococcus aureus
WP_053005248.1#120|Staphylococcus aureus
WP_230852042.1#69|Staphylococcus aureus
WP_160189991.1#96|Staphylococcus aureus
WP_154290506.1#152|Staphylococcus aureus
WP_216745732.1#6|Staphylococcus aureus
WP_249524812.1#1|Staphylococcus aureus
WP_113589264.1#8|Staphylococcus aureus
WP_165618745.1#26|Staphylococcus aureus
WP_113590375.1#27|Staphylococcus aureus
WP_141062233.1#25|Staphylococcus aureus
WP_001208922.1#22|Staphylococcus aureus
WP_064132079.1#18|Staphylococcus aureus
WP_312013423.1#17|Staphylococcus aureus
WP_205298771.1#23|Staphylococcus aureus
WP_165619758.1#74|Staphylococcus aureus
WP_113580790.1#68|Staphylococcus aureus
WP_113590253.1#49|Staphylococcus aureus
WP_072468356.1#15|Staphylococcus aureus
WP_234728030.1#14|Staphylococcus aureus
WP_072526490.1#9|Staphylococcus aureus
WP_274544019.1#16|Staphylococcus aureus
WP_049307735.1#7|Staphylococcus aureus
WP_268560092.1#13|Staphylococcus aureus
WP_000330443.1#167|Staphylococcus aureus subsp aureus USA300 TCH959
WP_262537462.1#63|Staphylococcus aureus
WP_252583659.1#181|Staphylococcus aureus
WP_301397077.1#5|Staphylococcus aureus
WP_070044204.1#2|Staphylococcus aureus
WP_281224548.1#4|Staphylococcus aureus
WP_258410990.1#3|Staphylococcus aureus
WP_301557592.1#21|Staphylococcus aureus
WP_071080448.1#12|Staphylococcus aureus
WP_070047672.1#10|Staphylococcus aureus
WP_252094375.1#121|Staphylococcus aureus
WP_250543457.1#138|Staphylococcus aureus
WP_147715355.1#100|Staphylococcus aureus
WP_250322217.1#132|Staphylococcus aureus
WP_031914721.1#92|Staphylococcus aureus VET0183R
WP_111090025.1#11|Staphylococcus aureus
WP_238608744.1#143|Staphylococcus aureus
WP_049296481.1#124|Staphylococcus aureus
WP_045177089.1#144|Staphylococcus aureus
WP_117215976.1#154|Staphylococcus aureus
WP_117434111.1#151|Staphylococcus aureus
WP_117203222.1#145|Staphylococcus aureus
WP_064128608.1#150|Staphylococcus aureus
WP_155977923.1#175|Staphylococcus aureus
WP_115305133.1#128|Staphylococcus aureus
WP_222637344.1#139|Staphylococcus aureus
WP_001208905.1#136|Staphylococcus aureus
WP_233642320.1#142|Staphylococcus aureus
WP_196931808.1#135|Staphylococcus aureus
WP_069994155.1#141|Staphylococcus aureus
WP_086153280.1#125|Staphylococcus aureus
WP_258413703.1#168|Staphylococcus aureus
WP_104011172.1#161|Staphylococcus aureus
WP_029550030.1#157|Staphylococcus aureus
WP_107366818.1#156|Staphylococcus aureus
WP_061819853.1#162|Staphylococcus aureus
WP_072435036.1#160|Staphylococcus aureus
WP_001208906.1#159|Staphylococcus aureus subsp aureus 21305
WP_062909758.1#164|Staphylococcus aureus
WP_258416576.1#165|Staphylococcus aureus
WP_258412434.1#163|Staphylococcus aureus
WP_262611027.1#166|Staphylococcus aureus
WP_070046246.1#137|Staphylococcus aureus
WP_070043487.1#123|Staphylococcus aureus
WP_070064300.1#130|Staphylococcus aureus
WP_001208894.1#126|Staphylococcus aureus
WP_061735479.1#129|Staphylococcus aureus
WP_252549628.1#131|Staphylococcus aureus
WP_070007303.1#127|Staphylococcus aureus
WP_115390782.1#32|Staphylococcus aureus
WP_072496824.1#115|Staphylococcus aureus
WP_061838930.1#119|Staphylococcus aureus
WP_001208890.1#122|Staphylococcus aureus
WP_216728190.1#98|Staphylococcus aureus
WP_283588743.1#81|Staphylococcus aureus
WP_267830219.1#91|Staphylococcus aureus
WP_103212144.1#94|Staphylococcus aureus
WP_001208892.1#87|Staphylococcus aureus MISS6103
WP_117207340.1#117|Staphylococcus aureus
WP_165621829.1#78|Staphylococcus aureus
WP_270758916.1#111|Staphylococcus aureus
WP_260633830.1#113|Staphylococcus aureus
WP_198987419.1#116|Staphylococcus aureus
WP_259696468.1#88|Staphylococcus aureus
WP_234863418.1#101|Staphylococcus aureus
WP_084985139.1#106|Staphylococcus aureus
WP_117201024.1#86|Staphylococcus aureus
WP_086098487.1#105|Staphylococcus aureus
WP_199004398.1#104|Staphylococcus aureus
WP_154289154.1#108|Staphylococcus aureus
WP_099143779.1#99|Staphylococcus aureus
WP_261933994.1#109|Staphylococcus aureus
WP_283591143.1#53|Staphylococcus aureus
WP_271287700.1#82|Staphylococcus aureus
WP_072527585.1#85|Staphylococcus aureus
WP_198996452.1#102|Staphylococcus aureus
WP_001208893.1#90|Staphylococcus aureus
WP_234722849.1#95|Staphylococcus aureus
WP_049314019.1#97|Staphylococcus aureus
WP_162636527.1#89|Staphylococcus aureus
WP_154269472.1#84|Staphylococcus aureus
WP_031881528.1#112|Staphylococcus aureus AMMC6111
WP_064133281.1#103|Staphylococcus aureus
WP_001208919.1#29|Staphylococcus aureus subsp aureus VRS3a
WP_031859900.1#176|Staphylococcus aureus M0935
WP_031825044.1#188|Staphylococcus aureus M0452
WP_182045054.1#62|Staphylococcus aureus
WP_031795685.1#179|Staphylococcus aureus M0271
WP_001208917.1#24|Staphylococcus aureus H81901
WP_182055449.1#50|Staphylococcus aureus
WP_031794573.1#199|Staphylococcus aureus M0989
WP_031877219.1#189|Staphylococcus aureus M1156
WP_031832642.1#200|Staphylococcus aureus M0840
WP_031863209.1#198|Staphylococcus aureus M1018
WP_216749478.1#146|Staphylococcus aureus
WP_031808847.1#169|Staphylococcus aureus M1080
WP_031834419.1#170|Staphylococcus aureus M0598
WP_049318440.1#45|Staphylococcus aureus
WP_103183862.1#19|Staphylococcus aureus
WP_224132397.1#148|Staphylococcus aureus
WP_095255644.1#155|Staphylococcus aureus
WP_031878340.1#178|Staphylococcus aureus M0617
WP_031895930.1#177|Staphylococcus aureus M0808
WP_031887344.1#172|Staphylococcus aureus M0653
WP_031834070.1#173|Staphylococcus aureus M0508
WP_223223677.1#153|Staphylococcus aureus M0656
WP_031896026.1#171|Staphylococcus aureus M0809
WP_103147856.1#67|Staphylococcus aureus
WP_031793823.1#28|Staphylococcus aureus DAR5852
WP_086045059.1#38|Staphylococcus aureus
WP_312010534.1#114|Staphylococcus aureus
WP_031810960.1#51|Staphylococcus aureus M0435
WP_224800206.1#37|Staphylococcus aureus
WP_190338080.1#107|Staphylococcus aureus
WP_049879846.1#197|Staphylococcus aureus M0657
WP_049279118.1#83|Staphylococcus aureus
WP_274894661.1#79|Staphylococcus aureus
WP_111033032.1#60|Staphylococcus aureus
WP_001208918.1#42|Staphylococcus aureus M0298
WP_031825732.1#149|Staphylococcus aureus M0333
WP_031878262.1#174|Staphylococcus aureus M0612
WP_064133378.1#40|Staphylococcus aureus
WP_218468907.1#158|Staphylococcus aureus
WP_224077684.1#31|Staphylococcus aureus
WP_049882909.1#180|Staphylococcus aureus M0376
WP_086040561.1#73|Staphylococcus aureus
WP_086097212.1#76|Staphylococcus aureus
WP_232044238.1#77|Staphylococcus aureus
WP_001208920.1#134|Staphylococcus aureus subsp aureus IS 122
WP_070064200.1#59|Staphylococcus aureus
WP_260645050.1#75|Staphylococcus aureus
WP_114283425.1#110|Staphylococcus aureus
WP_145373910.1#30|Staphylococcus aureus
WP_031906458.1#72|Staphylococcus aureus SJUD6053
WP_086032581.1#118|Staphylococcus aureus
WP_117374314.1#39|Staphylococcus aureus
WP_111071215.1#93|Staphylococcus aureus
WP_064305721.1#80|Staphylococcus aureus
WP_233232812.1#46|Staphylococcus aureus
WP_176320916.1#34|Staphylococcus aureus
WP_103151719.1#66|Staphylococcus aureus
WP_225511228.1#57|Staphylococcus aureus
WP_267810978.1#33|Staphylococcus aureus
WP_238609917.1#43|Staphylococcus aureus
WP_031879712.1#64|Staphylococcus aureus
WP_250045062.1#133|Staphylococcus aureus
WP_279524263.1#36|Staphylococcus aureus
WP_103143397.1#58|Staphylococcus aureus
WP_095284808.1#41|Staphylococcus aureus
WP_111711090.1#55|Staphylococcus aureus
WP_269634158.1#44|Staphylococcus aureus
WP_103149213.1#48|Staphylococcus aureus
WP_001638850.1#47|Staphylococcus aureus M0399
WP_001208916.1#52|Staphylococcus aureus subsp aureus CIG1165
WP_031905719.1#56|Staphylococcus aureus SJOS6016
WP_061839331.1#54|Staphylococcus aureus
WP_064131152.1#70|Staphylococcus aureus
WP_031862898.1#61|Staphylococcus aureus T57397
WP_064275991.1#71|Staphylococcus aureus
WP_031793476.1#65|Staphylococcus aureus HBHO6042
WP_289810327.1#35|Staphylococcus aureus
