# Supplementary material for: A type VII-secreted lipase toxin with reverse domain arrangement
Source: Nat Commun. 2023 Dec 19;14:8438. doi: 10.1038/s41467-023-44221-y (PMC10730906; doi:10.1038/s41467-023-44221-y)
Supplement: Supplementary file 6 — Source Data [file 41467_2023_44221_MOESM6_ESM.zip › Tsl1 distribution raw/lpl0 3/FlaGs_output/results_TreeOrder_output.pdf]

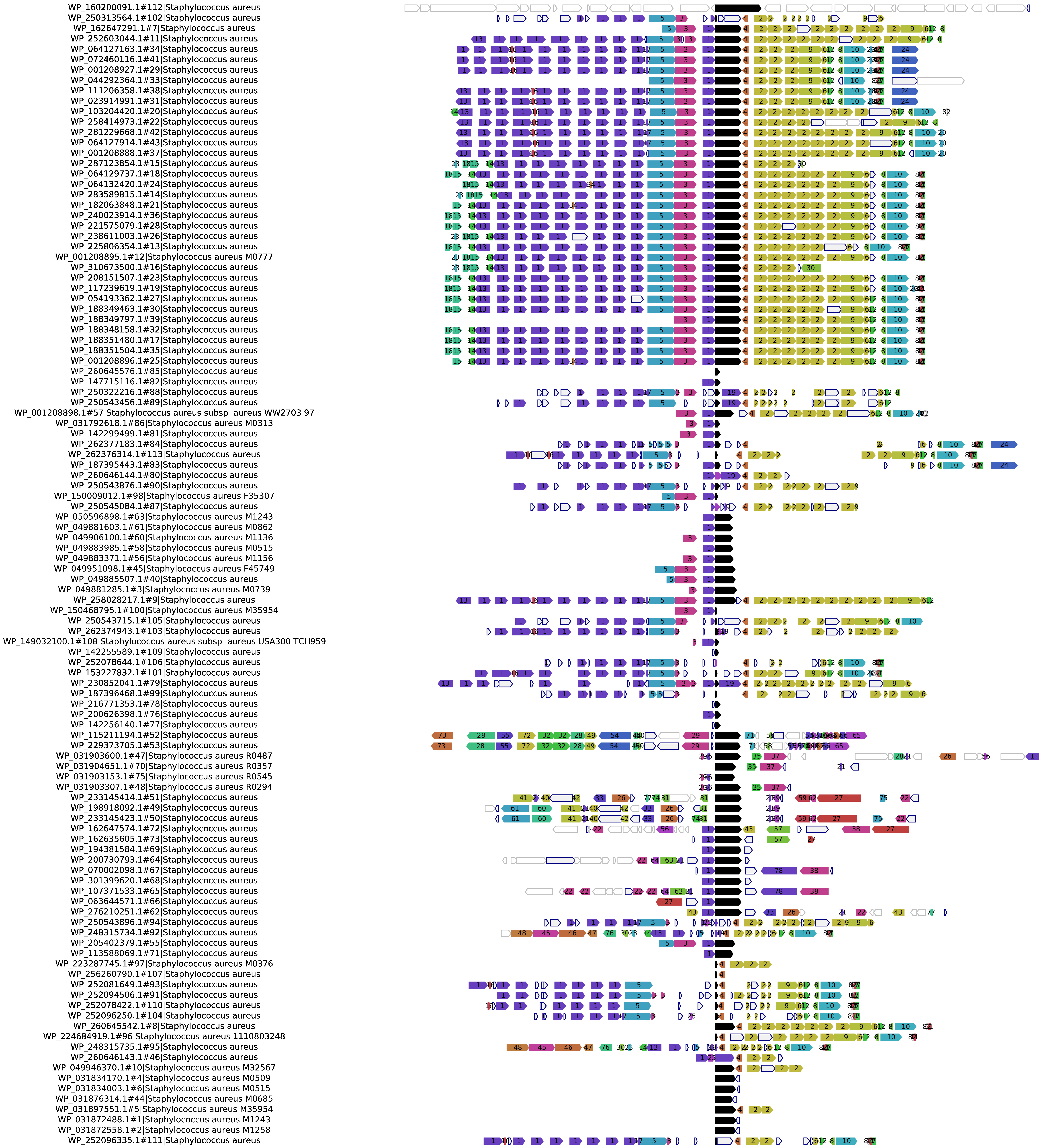
WP_160200091.1#112|Staphylococcus aureus
WP_250313564.1#102|Staphylococcus aureus
WP_162647291.1#7|Staphylococcus aureus
WP_252603044.1#11|Staphylococcus aureus
WP_064127163.1#34|Staphylococcus aureus
WP_072460116.1#41|Staphylococcus aureus
WP_001208927.1#29|Staphylococcus aureus
WP_044292364.1#33|Staphylococcus aureus
WP_111206358.1#38|Staphylococcus aureus
WP_023914991.1#31|Staphylococcus aureus
WP_103204420.1#20|Staphylococcus aureus
WP_258414973.1#22|Staphylococcus aureus
WP_281229668.1#42|Staphylococcus aureus
WP_064127914.1#43|Staphylococcus aureus
WP_001208888.1#37|Staphylococcus aureus
WP_287123854.1#15|Staphylococcus aureus
WP_064129737.1#18|Staphylococcus aureus
WP_064132420.1#24|Staphylococcus aureus
WP_283589815.1#14|Staphylococcus aureus
WP_182063848.1#21|Staphylococcus aureus
WP_240023914.1#36|Staphylococcus aureus
WP_221575079.1#28|Staphylococcus aureus
WP_238611003.1#26|Staphylococcus aureus
WP_225806354.1#13|Staphylococcus aureus
WP_001208895.1#12|Staphylococcus aureus M0777
WP_310673500.1#16|Staphylococcus aureus
WP_208151507.1#23|Staphylococcus aureus
WP_117239619.1#19|Staphylococcus aureus
WP_054193362.1#27|Staphylococcus aureus
WP_188349463.1#30|Staphylococcus aureus
WP_188349797.1#39|Staphylococcus aureus
WP_188348158.1#32|Staphylococcus aureus
WP_188351480.1#17|Staphylococcus aureus
WP_188351504.1#35|Staphylococcus aureus
WP_001208896.1#25|Staphylococcus aureus
WP_260645576.1#85|Staphylococcus aureus
WP_147715116.1#82|Staphylococcus aureus
WP_250322216.1#88|Staphylococcus aureus
WP_250543456.1#89|Staphylococcus aureus
WP_001208898.1#57|Staphylococcus aureus subsp aureus WW2703 97
WP_031792618.1#86|Staphylococcus aureus M0313
WP_142299499.1#81|Staphylococcus aureus
WP_262377183.1#84|Staphylococcus aureus
WP_262376314.1#113|Staphylococcus aureus
WP_187395443.1#83|Staphylococcus aureus
WP_260646144.1#80|Staphylococcus aureus
WP_250543876.1#90|Staphylococcus aureus
WP_150009012.1#98|Staphylococcus aureus F35307
WP_250545084.1#87|Staphylococcus aureus
WP_050596898.1#63|Staphylococcus aureus M1243
WP_049881603.1#61|Staphylococcus aureus M0862
WP_049906100.1#60|Staphylococcus aureus M1136
WP_049883985.1#58|Staphylococcus aureus M0515
WP_049883371.1#56|Staphylococcus aureus M1156
WP_049951098.1#45|Staphylococcus aureus F45749
WP_049885507.1#40|Staphylococcus aureus
WP_049881285.1#3|Staphylococcus aureus M0739
WP_258028217.1#9|Staphylococcus aureus
WP_150468795.1#100|Staphylococcus aureus M35954
WP_250543715.1#105|Staphylococcus aureus
WP_262374943.1#103|Staphylococcus aureus
WP_149032100.1#108|Staphylococcus aureus subsp aureus USA300 TCH959
WP_142255589.1#109|Staphylococcus aureus
WP_252078644.1#106|Staphylococcus aureus
WP_153227832.1#101|Staphylococcus aureus
WP_230852041.1#79|Staphylococcus aureus
WP_187396468.1#99|Staphylococcus aureus
WP_216771353.1#78|Staphylococcus aureus
WP_200626398.1#76|Staphylococcus aureus
WP_142256140.1#77|Staphylococcus aureus
WP_115211194.1#52|Staphylococcus aureus
WP_229373705.1#53|Staphylococcus aureus
WP_031903600.1#47|Staphylococcus aureus R0487
WP_031904651.1#70|Staphylococcus aureus R0357
WP_031903153.1#75|Staphylococcus aureus R0545
WP_031903307.1#48|Staphylococcus aureus R0294
WP_233145414.1#51|Staphylococcus aureus
WP_198918092.1#49|Staphylococcus aureus
WP_233145423.1#50|Staphylococcus aureus
WP_162647574.1#72|Staphylococcus aureus
WP_162635605.1#73|Staphylococcus aureus
WP_194381584.1#69|Staphylococcus aureus
WP_200730793.1#64|Staphylococcus aureus
WP_070002098.1#67|Staphylococcus aureus
WP_301399620.1#68|Staphylococcus aureus
WP_107371533.1#65|Staphylococcus aureus
WP_063644571.1#66|Staphylococcus aureus
WP_276210251.1#62|Staphylococcus aureus
WP_250543896.1#94|Staphylococcus aureus
WP_248315734.1#92|Staphylococcus aureus
WP_205402379.1#55|Staphylococcus aureus
WP_113588069.1#71|Staphylococcus aureus
WP_223287745.1#97|Staphylococcus aureus M0376
WP_256260790.1#107|Staphylococcus aureus
WP_252081649.1#93|Staphylococcus aureus
WP_252094506.1#91|Staphylococcus aureus
WP_252078422.1#110|Staphylococcus aureus
WP_252096250.1#104|Staphylococcus aureus
WP_260645542.1#8|Staphylococcus aureus
WP_224684919.1#96|Staphylococcus aureus 1110803248
WP_248315735.1#95|Staphylococcus aureus
WP_260646143.1#46|Staphylococcus aureus
WP_049946370.1#10|Staphylococcus aureus M32567
WP_031834170.1#4|Staphylococcus aureus M0509
WP_031834003.1#6|Staphylococcus aureus M0515
WP_031876314.1#44|Staphylococcus aureus M0685
WP_031897551.1#5|Staphylococcus aureus M35954
WP_031872488.1#1|Staphylococcus aureus M1243
WP_031872558.1#2|Staphylococcus aureus M1258
WP_252096335.1#111|Staphylococcus aureus
