# Supplementary material for: A type VII-secreted lipase toxin with reverse domain arrangement
Source: Nat Commun. 2023 Dec 19;14:8438. doi: 10.1038/s41467-023-44221-y (PMC10730906; doi:10.1038/s41467-023-44221-y)
Supplement: Supplementary file 6 — Source Data [file 41467_2023_44221_MOESM6_ESM.zip › Tsl1 distribution raw/lplI 1/FlaGs_output/results_TreeOrder_output.pdf]

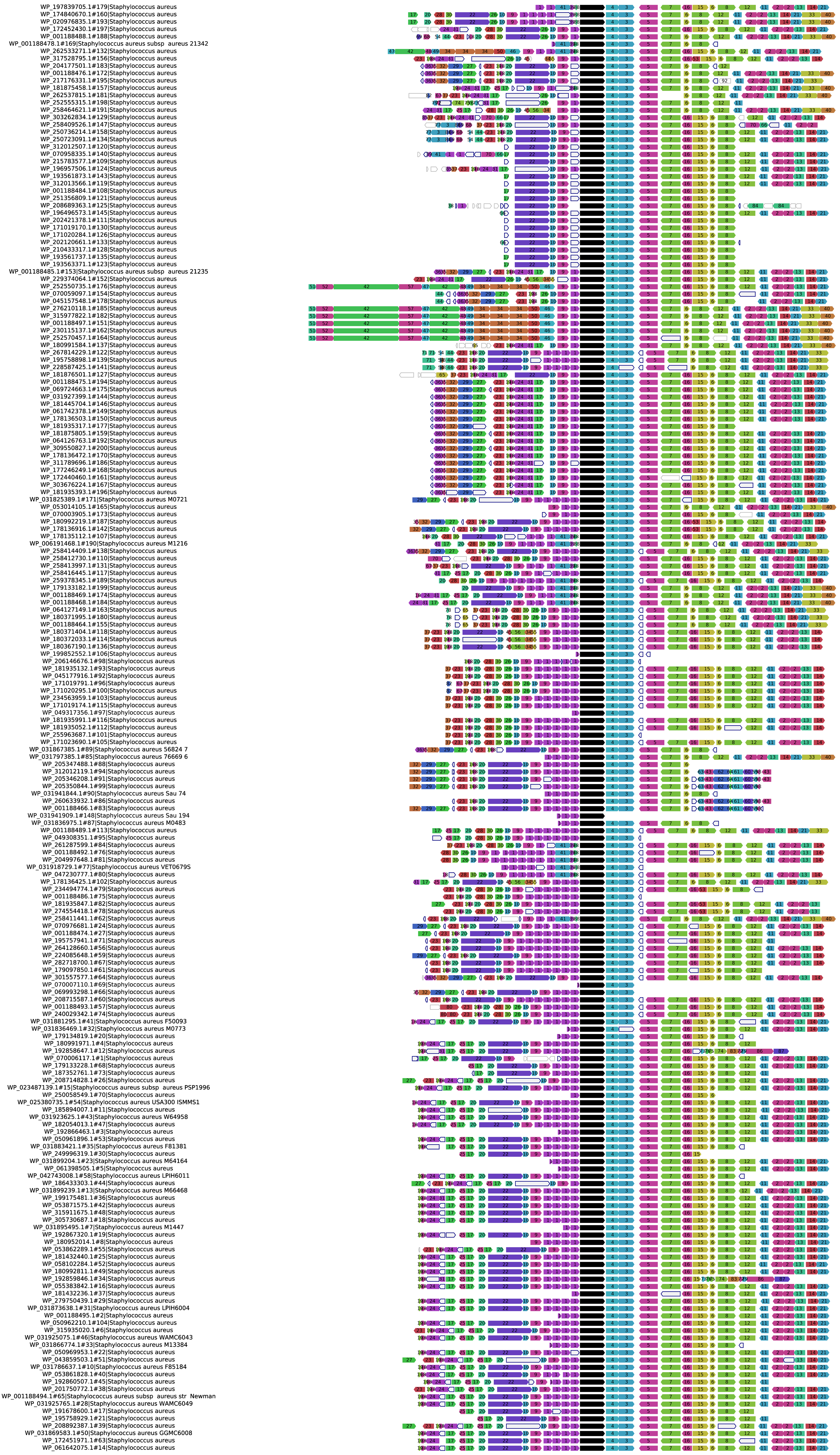

WP_197839705.1#179|Staphylococcus aureus
WP_174840670.1#160|Staphylococcus aureus
WP_020976835.1#193|Staphylococcus aureus
WP_172452430.1#197|Staphylococcus aureus
WP_001188488.1#188|Staphylococcus aureus
WP_001188478.1#169|Staphylococcus aureus subsp aureus 21342
WP_262533271.1#132|Staphylococcus aureus
WP_317528795.1#156|Staphylococcus aureus
WP_204177501.1#183|Staphylococcus aureus
WP_001188476.1#172|Staphylococcus aureus
WP_217176331.1#195|Staphylococcus aureus
WP_181875458.1#157|Staphylococcus aureus
WP_262537815.1#181|Staphylococcus aureus
WP_252555315.1#198|Staphylococcus aureus
WP_258464621.1#191|Staphylococcus aureus
WP_303262834.1#129|Staphylococcus aureus
WP_258409526.1#147|Staphylococcus aureus
WP_250736214.1#158|Staphylococcus aureus
WP_250723091.1#134|Staphylococcus aureus
WP_312012507.1#120|Staphylococcus aureus
WP_070958335.1#140|Staphylococcus aureus
WP_215783577.1#109|Staphylococcus aureus
WP_196957506.1#124|Staphylococcus aureus
WP_193561873.1#143|Staphylococcus aureus
WP_312013566.1#119|Staphylococcus aureus
WP_001188484.1#108|Staphylococcus aureus
WP_251356809.1#121|Staphylococcus aureus
WP_208689363.1#125|Staphylococcus aureus
WP_196496573.1#145|Staphylococcus aureus
WP_202421378.1#111|Staphylococcus aureus
WP_171019170.1#130|Staphylococcus aureus
WP_171020284.1#126|Staphylococcus aureus
WP_202120661.1#133|Staphylococcus aureus
WP_210433317.1#128|Staphylococcus aureus
WP_193561737.1#135|Staphylococcus aureus
WP_193563371.1#123|Staphylococcus aureus
WP_001188485.1#153|Staphylococcus aureus subsp aureus 21235
WP_229374064.1#152|Staphylococcus aureus
WP_252550735.1#176|Staphylococcus aureus
WP_070059097.1#154|Staphylococcus aureus
WP_045157548.1#178|Staphylococcus aureus
WP_276210118.1#185|Staphylococcus aureus
WP_315977822.1#182|Staphylococcus aureus
WP_001188497.1#151|Staphylococcus aureus
WP_230115137.1#162|Staphylococcus aureus
WP_252570457.1#164|Staphylococcus aureus
WP_180991584.1#137|Staphylococcus aureus
WP_267814229.1#122|Staphylococcus aureus
WP_195758898.1#139|Staphylococcus aureus
WP_228587425.1#141|Staphylococcus aureus
WP_181876501.1#127|Staphylococcus aureus
WP_001188475.1#194|Staphylococcus aureus
WP_069724663.1#175|Staphylococcus aureus
WP_031927399.1#144|Staphylococcus aureus
WP_181445704.1#146|Staphylococcus aureus
WP_061742378.1#149|Staphylococcus aureus
WP_178136503.1#150|Staphylococcus aureus
WP_181935317.1#177|Staphylococcus aureus
WP_181875805.1#159|Staphylococcus aureus
WP_064126763.1#192|Staphylococcus aureus
WP_309550827.1#200|Staphylococcus aureus
WP_178136472.1#170|Staphylococcus aureus
WP_311789696.1#186|Staphylococcus aureus
WP_177246249.1#168|Staphylococcus aureus
WP_172440460.1#161|Staphylococcus aureus
WP_303676224.1#167|Staphylococcus aureus
WP_181935393.1#196|Staphylococcus aureus
WP_031825389.1#171|Staphylococcus aureus M0721
WP_053014105.1#165|Staphylococcus aureus
WP_070003905.1#173|Staphylococcus aureus
WP_180992219.1#187|Staphylococcus aureus
WP_178136916.1#142|Staphylococcus aureus
WP_178135112.1#107|Staphylococcus aureus
WP_006191468.1#190|Staphylococcus aureus M1216
WP_258414409.1#138|Staphylococcus aureus
WP_258412730.1#110|Staphylococcus aureus
WP_258413997.1#131|Staphylococcus aureus
WP_258416445.1#117|Staphylococcus aureus
WP_259378345.1#189|Staphylococcus aureus
WP_179133182.1#199|Staphylococcus aureus
WP_001188469.1#174|Staphylococcus aureus
WP_001188468.1#184|Staphylococcus aureus
WP_064127149.1#163|Staphylococcus aureus
WP_180371995.1#180|Staphylococcus aureus
WP_001188464.1#155|Staphylococcus aureus
WP_180371404.1#118|Staphylococcus aureus
WP_180372033.1#114|Staphylococcus aureus
WP_180367190.1#136|Staphylococcus aureus
WP_199852552.1#106|Staphylococcus aureus
WP_206146676.1#98|Staphylococcus aureus
WP_181935132.1#93|Staphylococcus aureus
WP_045177916.1#92|Staphylococcus aureus
WP_171019791.1#96|Staphylococcus aureus
WP_171020295.1#100|Staphylococcus aureus
WP_234563959.1#103|Staphylococcus aureus
WP_171019174.1#115|Staphylococcus aureus
WP_049317356.1#97|Staphylococcus aureus
WP_181935991.1#116|Staphylococcus aureus
WP_181935052.1#112|Staphylococcus aureus
WP_255963687.1#101|Staphylococcus aureus
WP_171023690.1#105|Staphylococcus aureus
WP_031867385.1#89|Staphylococcus aureus 56824 7
WP_031797385.1#85|Staphylococcus aureus 76669 6
WP_205347488.1#88|Staphylococcus aureus
WP_312012119.1#94|Staphylococcus aureus
WP_205346208.1#91|Staphylococcus aureus
WP_205350844.1#99|Staphylococcus aureus
WP_031941844.1#90|Staphylococcus aureus Sau 74
WP_260633932.1#86|Staphylococcus aureus
WP_001188466.1#83|Staphylococcus aureus
WP_031941909.1#148|Staphylococcus aureus Sau 194
WP_031836975.1#87|Staphylococcus aureus M0483
WP_001188489.1#113|Staphylococcus aureus
WP_049308351.1#95|Staphylococcus aureus
WP_261287599.1#84|Staphylococcus aureus
WP_001188492.1#76|Staphylococcus aureus
WP_204997648.1#81|Staphylococcus aureus
WP_031918729.1#77|Staphylococcus aureus VET0679S
WP_047230777.1#80|Staphylococcus aureus
WP_178136425.1#102|Staphylococcus aureus
WP_234494774.1#79|Staphylococcus aureus
WP_001188486.1#75|Staphylococcus aureus
WP_181935847.1#82|Staphylococcus aureus
WP_274554418.1#78|Staphylococcus aureus
WP_258411441.1#62|Staphylococcus aureus
WP_070976681.1#24|Staphylococcus aureus
WP_001188474.1#27|Staphylococcus aureus
WP_195757941.1#71|Staphylococcus aureus
WP_264128660.1#56|Staphylococcus aureus
WP_224085648.1#59|Staphylococcus aureus
WP_282718700.1#67|Staphylococcus aureus
WP_179097850.1#61|Staphylococcus aureus
WP_301557577.1#64|Staphylococcus aureus
WP_070007110.1#69|Staphylococcus aureus
WP_069993298.1#66|Staphylococcus aureus
WP_208715587.1#60|Staphylococcus aureus
WP_001188493.1#57|Staphylococcus aureus
WP_240029342.1#74|Staphylococcus aureus
WP_031881295.1#41|Staphylococcus aureus F50093
WP_031836469.1#32|Staphylococcus aureus M0773
WP_179134819.1#20|Staphylococcus aureus
WP_180991971.1#4|Staphylococcus aureus
WP_192858647.1#12|Staphylococcus aureus
WP_070006117.1#1|Staphylococcus aureus
WP_179133228.1#68|Staphylococcus aureus
WP_187352761.1#73|Staphylococcus aureus
WP_208714828.1#26|Staphylococcus aureus
WP_023487139.1#15|Staphylococcus aureus subsp aureus PSP1996
WP_250058549.1#70|Staphylococcus aureus
WP_025380735.1#54|Staphylococcus aureus USA300 ISMMS1
WP_185894007.1#11|Staphylococcus aureus
WP_031923625.1#43|Staphylococcus aureus W64958
WP_182054013.1#47|Staphylococcus aureus
WP_192866463.1#3|Staphylococcus aureus
WP_050961896.1#53|Staphylococcus aureus
WP_031883421.1#35|Staphylococcus aureus F81381
WP_249996319.1#30|Staphylococcus aureus
WP_031899204.1#23|Staphylococcus aureus M64164
WP_061398505.1#5|Staphylococcus aureus
WP_042743008.1#58|Staphylococcus aureus LPIH6011
WP_186433303.1#44|Staphylococcus aureus
WP_031899239.1#13|Staphylococcus aureus M66468
WP_199175481.1#36|Staphylococcus aureus
WP_053871575.1#42|Staphylococcus aureus
WP_315911675.1#48|Staphylococcus aureus
WP_305730687.1#18|Staphylococcus aureus
WP_031895495.1#7|Staphylococcus aureus M1447
WP_192867320.1#19|Staphylococcus aureus
WP_180952014.1#8|Staphylococcus aureus
WP_053862289.1#55|Staphylococcus aureus
WP_181432440.1#25|Staphylococcus aureus
WP_058102284.1#52|Staphylococcus aureus
WP_180992811.1#49|Staphylococcus aureus
WP_192859846.1#34|Staphylococcus aureus
WP_055383842.1#16|Staphylococcus aureus
WP_181432236.1#37|Staphylococcus aureus
WP_279750439.1#29|Staphylococcus aureus
WP_031873638.1#31|Staphylococcus aureus LPIH6004
WP_001188495.1#2|Staphylococcus aureus
WP_050962210.1#104|Staphylococcus aureus
WP_315935020.1#6|Staphylococcus aureus
WP_031925075.1#46|Staphylococcus aureus WAMC6043
WP_031866774.1#33|Staphylococcus aureus M13384
WP_050969953.1#22|Staphylococcus aureus
WP_043859503.1#51|Staphylococcus aureus
WP_031786637.1#10|Staphylococcus aureus F85184
WP_053861828.1#40|Staphylococcus aureus
WP_192860507.1#45|Staphylococcus aureus
WP_201750772.1#38|Staphylococcus aureus
WP_001188494.1#65|Staphylococcus aureus subsp aureus str Newman
WP_031925765.1#28|Staphylococcus aureus WAMC6049
WP_191678600.1#17|Staphylococcus aureus
WP_195758929.1#21|Staphylococcus aureus
WP_208892387.1#39|Staphylococcus aureus
WP_031869583.1#50|Staphylococcus aureus GGMC6008
WP_172451971.1#63|Staphylococcus aureus
WP_061642075.1#14|Staphylococcus aureus
