# Supplementary material for: A type VII-secreted lipase toxin with reverse domain arrangement
Source: Nat Commun. 2023 Dec 19;14:8438. doi: 10.1038/s41467-023-44221-y (PMC10730906; doi:10.1038/s41467-023-44221-y)
Supplement: Supplementary file 6 — Source Data [file 41467_2023_44221_MOESM6_ESM.zip › Tsl1 distribution raw/lplI 2/FlaGs_output/results_operon.pdf]

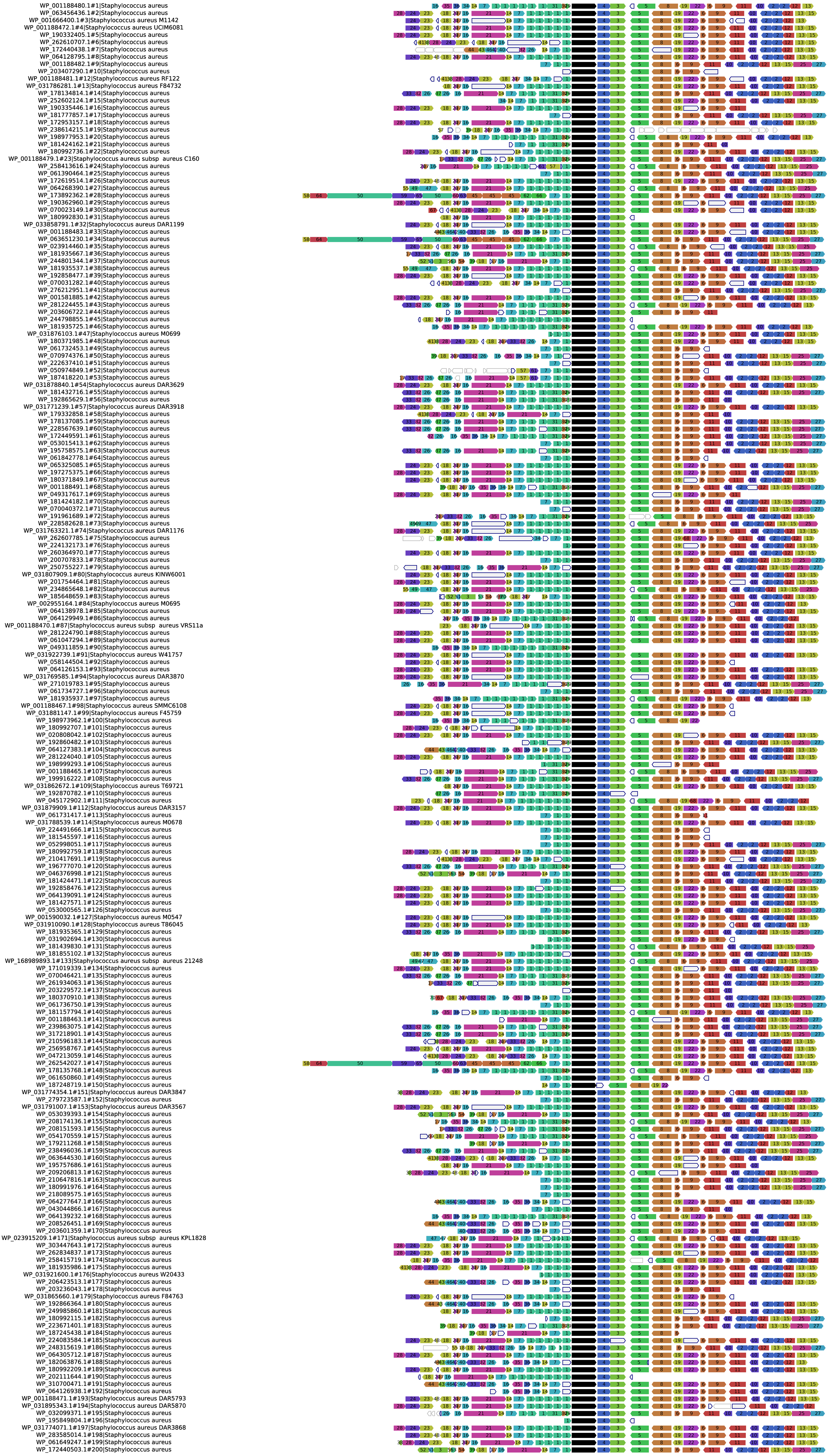

WP_001188480.1#1|Staphylococcus aureus
WP_063456436.1#2|Staphylococcus aureus
WP_001666400.1#3|Staphylococcus aureus M1142
WP_001188472.1#4|Staphylococcus aureus UCIM6081
WP_190332405.1#5|Staphylococcus aureus
WP_262610707.1#6|Staphylococcus aureus
WP_172440438.1#7|Staphylococcus aureus
WP_064128795.1#8|Staphylococcus aureus
WP_001188482.1#9|Staphylococcus aureus
WP_203407290.1#10|Staphylococcus aureus
WP_001188481.1#12|Staphylococcus aureus RF122
WP_031786281.1#13|Staphylococcus aureus F84732
WP_178134814.1#14|Staphylococcus aureus
WP_252602124.1#15|Staphylococcus aureus
WP_190335446.1#16|Staphylococcus aureus
WP_181777857.1#17|Staphylococcus aureus
WP_172953157.1#18|Staphylococcus aureus
WP_238614215.1#19|Staphylococcus aureus
WP_198977953.1#20|Staphylococcus aureus
WP_181424162.1#21|Staphylococcus aureus
WP_180992736.1#22|Staphylococcus aureus
WP_001188479.1#23|Staphylococcus aureus subsp aureus C160
WP_258413616.1#24|Staphylococcus aureus
WP_061390464.1#25|Staphylococcus aureus
WP_172619514.1#26|Staphylococcus aureus
WP_064268390.1#27|Staphylococcus aureus
WP_173892362.1#28|Staphylococcus aureus
WP_190362960.1#29|Staphylococcus aureus
WP_070023149.1#30|Staphylococcus aureus
WP_180992830.1#31|Staphylococcus aureus
WP_033858791.1#32|Staphylococcus aureus DAR1199
WP_001188483.1#33|Staphylococcus aureus
WP_063651230.1#34|Staphylococcus aureus
WP_023914460.1#35|Staphylococcus aureus
WP_181935667.1#36|Staphylococcus aureus
WP_244801344.1#37|Staphylococcus aureus
WP_181935537.1#38|Staphylococcus aureus
WP_192858477.1#39|Staphylococcus aureus
WP_070031282.1#40|Staphylococcus aureus
WP_276212951.1#41|Staphylococcus aureus
WP_001581885.1#42|Staphylococcus aureus
WP_281224455.1#43|Staphylococcus aureus
WP_203606722.1#44|Staphylococcus aureus
WP_244798855.1#45|Staphylococcus aureus
WP_181935725.1#46|Staphylococcus aureus
WP_031876103.1#47|Staphylococcus aureus M0699
WP_180371985.1#48|Staphylococcus aureus
WP_061732453.1#49|Staphylococcus aureus
WP_070974376.1#50|Staphylococcus aureus
WP_222637410.1#51|Staphylococcus aureus
WP_050974849.1#52|Staphylococcus aureus
WP_187418220.1#53|Staphylococcus aureus
WP_031878840.1#54|Staphylococcus aureus DAR3629
WP_181432716.1#55|Staphylococcus aureus
WP_192865629.1#56|Staphylococcus aureus
WP_031771239.1#57|Staphylococcus aureus DAR3918
WP_179332858.1#58|Staphylococcus aureus
WP_178137085.1#59|Staphylococcus aureus
WP_228567639.1#60|Staphylococcus aureus
WP_172449591.1#61|Staphylococcus aureus
WP_053015413.1#62|Staphylococcus aureus
WP_195758575.1#63|Staphylococcus aureus
WP_061842778.1#64|Staphylococcus aureus
WP_065325085.1#65|Staphylococcus aureus
WP_197275375.1#66|Staphylococcus aureus
WP_180371849.1#67|Staphylococcus aureus
WP_001188491.1#68|Staphylococcus aureus
WP_049317617.1#69|Staphylococcus aureus
WP_181424182.1#70|Staphylococcus aureus
WP_070040372.1#71|Staphylococcus aureus
WP_191961689.1#72|Staphylococcus aureus
WP_228582628.1#73|Staphylococcus aureus
WP_031763321.1#74|Staphylococcus aureus DAR1176
WP_262607785.1#75|Staphylococcus aureus
WP_224132173.1#76|Staphylococcus aureus
WP_260364970.1#77|Staphylococcus aureus
WP_200707833.1#78|Staphylococcus aureus
WP_250755227.1#79|Staphylococcus aureus
WP_031807909.1#80|Staphylococcus aureus KINW6001
WP_201754464.1#81|Staphylococcus aureus
WP_234865648.1#82|Staphylococcus aureus
WP_185648659.1#83|Staphylococcus aureus
WP_002955164.1#84|Staphylococcus aureus M0695
WP_064138978.1#85|Staphylococcus aureus
WP_064129949.1#86|Staphylococcus aureus
WP_001188470.1#87|Staphylococcus aureus subsp aureus VRS11a
WP_281224790.1#88|Staphylococcus aureus
WP_061047294.1#89|Staphylococcus aureus
WP_049311859.1#90|Staphylococcus aureus
WP_031922739.1#91|Staphylococcus aureus W41757
WP_058144504.1#92|Staphylococcus aureus
WP_064126153.1#93|Staphylococcus aureus
WP_031769585.1#94|Staphylococcus aureus DAR3870
WP_271019783.1#95|Staphylococcus aureus
WP_061734727.1#96|Staphylococcus aureus
WP_181935937.1#97|Staphylococcus aureus
WP_001188467.1#98|Staphylococcus aureus SMMC6108
WP_031881147.1#99|Staphylococcus aureus F45759
WP_198973962.1#100|Staphylococcus aureus
WP_180992707.1#101|Staphylococcus aureus
WP_020808042.1#102|Staphylococcus aureus
WP_192860482.1#103|Staphylococcus aureus
WP_064127383.1#104|Staphylococcus aureus
WP_281224040.1#105|Staphylococcus aureus
WP_198999293.1#106|Staphylococcus aureus
WP_001188465.1#107|Staphylococcus aureus
WP_199916222.1#108|Staphylococcus aureus
WP_031862672.1#109|Staphylococcus aureus T69721
WP_192870782.1#110|Staphylococcus aureus
WP_045172902.1#111|Staphylococcus aureus
WP_031879909.1#112|Staphylococcus aureus DAR3157
WP_061731417.1#113|Staphylococcus aureus
WP_031788539.1#114|Staphylococcus aureus M0678
WP_224491666.1#115|Staphylococcus aureus
WP_181545597.1#116|Staphylococcus aureus
WP_052998051.1#117|Staphylococcus aureus
WP_180992759.1#118|Staphylococcus aureus
WP_210417691.1#119|Staphylococcus aureus
WP_196777070.1#120|Staphylococcus aureus
WP_046376998.1#121|Staphylococcus aureus
WP_181424471.1#122|Staphylococcus aureus
WP_192858476.1#123|Staphylococcus aureus
WP_064139091.1#124|Staphylococcus aureus
WP_181427571.1#125|Staphylococcus aureus
WP_053000565.1#126|Staphylococcus aureus
WP_001590032.1#127|Staphylococcus aureus M0547
WP_031910090.1#128|Staphylococcus aureus T86045
WP_181935365.1#129|Staphylococcus aureus
WP_031902694.1#130|Staphylococcus aureus
WP_181439830.1#131|Staphylococcus aureus
WP_181855102.1#132|Staphylococcus aureus
WP_168989893.1#133|Staphylococcus aureus subsp aureus 21248
WP_171019339.1#134|Staphylococcus aureus
WP_070046421.1#135|Staphylococcus aureus
WP_261934063.1#136|Staphylococcus aureus
WP_203229572.1#137|Staphylococcus aureus
WP_180370910.1#138|Staphylococcus aureus
WP_061736750.1#139|Staphylococcus aureus
WP_181157794.1#140|Staphylococcus aureus
WP_001188463.1#141|Staphylococcus aureus
WP_239863075.1#142|Staphylococcus aureus
WP_317218901.1#143|Staphylococcus aureus
WP_210596183.1#144|Staphylococcus aureus
WP_256958767.1#145|Staphylococcus aureus
WP_047213059.1#146|Staphylococcus aureus
WP_262542027.1#147|Staphylococcus aureus
WP_178135768.1#148|Staphylococcus aureus
WP_061650860.1#149|Staphylococcus aureus
WP_187248719.1#150|Staphylococcus aureus
WP_031774354.1#151|Staphylococcus aureus DAR3847
WP_279723587.1#152|Staphylococcus aureus
WP_031791007.1#153|Staphylococcus aureus DAR3567
WP_053039393.1#154|Staphylococcus aureus
WP_208174136.1#155|Staphylococcus aureus
WP_208151593.1#156|Staphylococcus aureus
WP_054170559.1#157|Staphylococcus aureus
WP_179211268.1#158|Staphylococcus aureus
WP_238496036.1#159|Staphylococcus aureus
WP_063644530.1#160|Staphylococcus aureus
WP_195757686.1#161|Staphylococcus aureus
WP_209206813.1#162|Staphylococcus aureus
WP_210647816.1#163|Staphylococcus aureus
WP_180991976.1#164|Staphylococcus aureus
WP_218089575.1#165|Staphylococcus aureus
WP_064277647.1#166|Staphylococcus aureus
WP_043044866.1#167|Staphylococcus aureus
WP_064139232.1#168|Staphylococcus aureus
WP_208526451.1#169|Staphylococcus aureus
WP_203601359.1#170|Staphylococcus aureus
WP_023915209.1#171|Staphylococcus aureus subsp aureus KPL1828
WP_303447643.1#172|Staphylococcus aureus
WP_262834837.1#173|Staphylococcus aureus
WP_258415719.1#174|Staphylococcus aureus
WP_181935986.1#175|Staphylococcus aureus
WP_031921600.1#176|Staphylococcus aureus W20433
WP_206423513.1#177|Staphylococcus aureus
WP_203236043.1#178|Staphylococcus aureus
WP_031865660.1#179|Staphylococcus aureus F84763
WP_192866364.1#180|Staphylococcus aureus
WP_249985860.1#181|Staphylococcus aureus
WP_180992115.1#182|Staphylococcus aureus
WP_223671401.1#183|Staphylococcus aureus
WP_187245438.1#184|Staphylococcus aureus
WP_224083584.1#185|Staphylococcus aureus
WP_248315619.1#186|Staphylococcus aureus
WP_064305712.1#187|Staphylococcus aureus
WP_182063876.1#188|Staphylococcus aureus
WP_180992209.1#189|Staphylococcus aureus
WP_202111644.1#190|Staphylococcus aureus
WP_310700471.1#191|Staphylococcus aureus
WP_064126938.1#192|Staphylococcus aureus
WP_001188471.1#193|Staphylococcus aureus DAR5793
WP_031895343.1#194|Staphylococcus aureus DAR5870
WP_032099371.1#195|Staphylococcus aureus
WP_195849804.1#196|Staphylococcus aureus
WP_031774071.1#197|Staphylococcus aureus DAR3868
WP_283585014.1#198|Staphylococcus aureus
WP_061649247.1#199|Staphylococcus aureus
WP_172440503.1#200|Staphylococcus aureus
