# Supplementary material for: A type VII-secreted lipase toxin with reverse domain arrangement
Source: Nat Commun. 2023 Dec 19;14:8438. doi: 10.1038/s41467-023-44221-y (PMC10730906; doi:10.1038/s41467-023-44221-y)
Supplement: Supplementary file 6 — Source Data [file 41467_2023_44221_MOESM6_ESM.zip › Tsl1 distribution raw/lplI 3/FlaGs_output/results_operon.pdf]

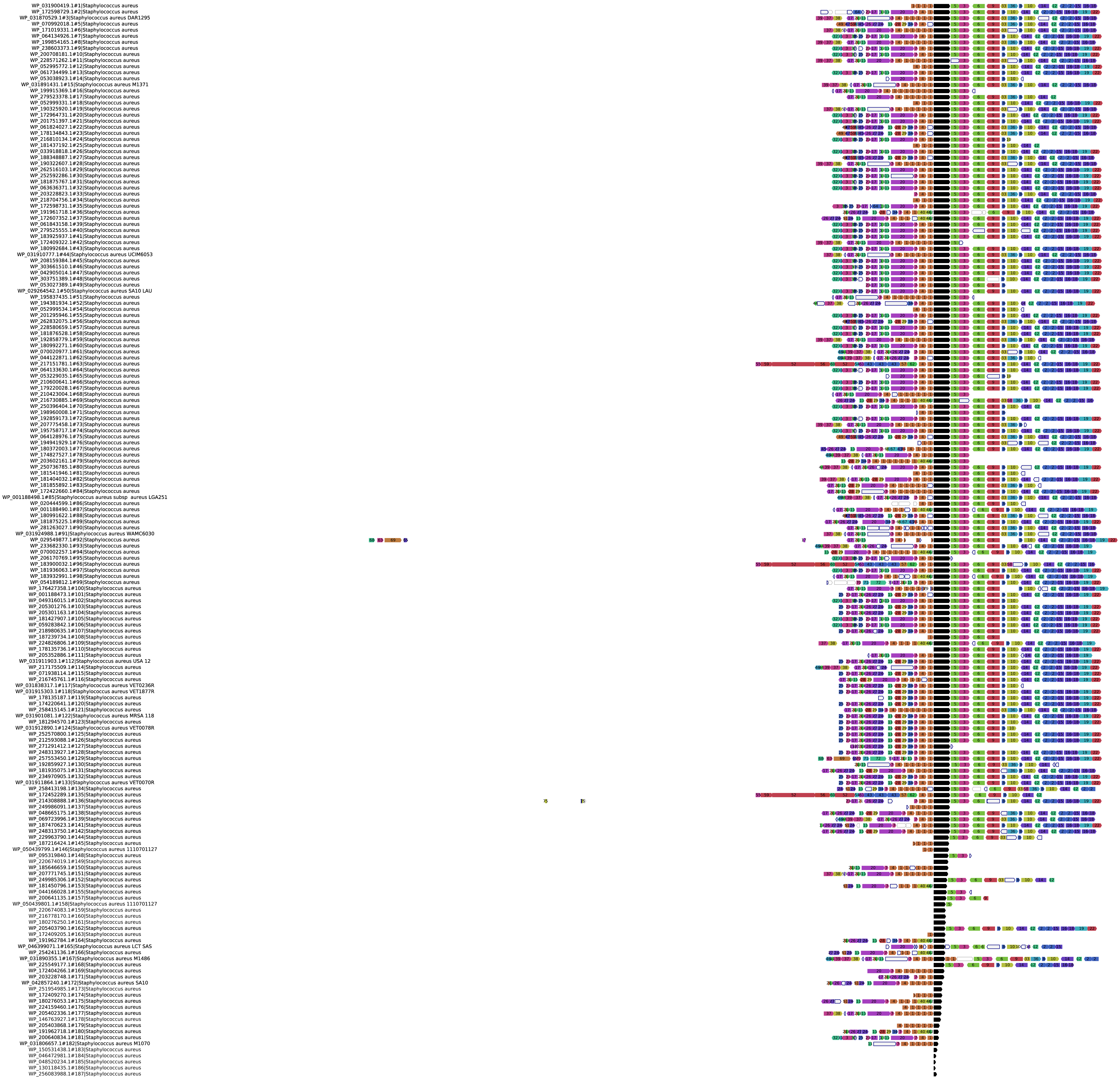
WP_031900419.1#1|Staphylococcus aureus
WP_172598729.1#2|Staphylococcus aureus
WP_031870529.1#3|Staphylococcus aureus DAR1295
WP_070992018.1#5|Staphylococcus aureus
WP_171019331.1#6|Staphylococcus aureus
WP_064134926.1#7|Staphylococcus aureus
WP_199854165.1#8|Staphylococcus aureus
WP_238603373.1#9|Staphylococcus aureus
WP_200708181.1#10|Staphylococcus aureus
WP_228571262.1#11|Staphylococcus aureus
WP_052995772.1#12|Staphylococcus aureus
WP_061734499.1#13|Staphylococcus aureus
WP_053038923.1#14|Staphylococcus aureus
WP_031891431.1#15|Staphylococcus aureus M1371
WP_199915369.1#16|Staphylococcus aureus
WP_279523378.1#17|Staphylococcus aureus
WP_052999331.1#18|Staphylococcus aureus
WP_190325920.1#19|Staphylococcus aureus
WP_172964731.1#20|Staphylococcus aureus
WP_201751397.1#21|Staphylococcus aureus
WP_061824027.1#22|Staphylococcus aureus
WP_178134843.1#23|Staphylococcus aureus
WP_216810134.1#24|Staphylococcus aureus
WP_181437192.1#25|Staphylococcus aureus
WP_033918818.1#26|Staphylococcus aureus
WP_188348887.1#27|Staphylococcus aureus
WP_190322607.1#28|Staphylococcus aureus
WP_262516103.1#29|Staphylococcus aureus
WP_252592286.1#30|Staphylococcus aureus
WP_181875767.1#31|Staphylococcus aureus
WP_063636371.1#32|Staphylococcus aureus
WP_203228823.1#33|Staphylococcus aureus
WP_218704756.1#34|Staphylococcus aureus
WP_172598731.1#35|Staphylococcus aureus
WP_191961718.1#36|Staphylococcus aureus
WP_172607352.1#37|Staphylococcus aureus
WP_061843158.1#39|Staphylococcus aureus
WP_279525555.1#40|Staphylococcus aureus
WP_183925937.1#41|Staphylococcus aureus
WP_172409322.1#42|Staphylococcus aureus
WP_180992684.1#43|Staphylococcus aureus
WP_031910777.1#44|Staphylococcus aureus UCIM6053
WP_208159384.1#45|Staphylococcus aureus
WP_303661510.1#46|Staphylococcus aureus
WP_042905014.1#47|Staphylococcus aureus
WP_303751389.1#48|Staphylococcus aureus
WP_053027389.1#49|Staphylococcus aureus
WP_029264542.1#50|Staphylococcus aureus SA10 LAU
WP_195837435.1#51|Staphylococcus aureus
WP_194381934.1#52|Staphylococcus aureus
WP_052999534.1#54|Staphylococcus aureus
WP_201295946.1#55|Staphylococcus aureus
WP_262832075.1#56|Staphylococcus aureus
WP_228580659.1#57|Staphylococcus aureus
WP_181876528.1#58|Staphylococcus aureus
WP_192858779.1#59|Staphylococcus aureus
WP_180992271.1#60|Staphylococcus aureus
WP_070020977.1#61|Staphylococcus aureus
WP_044122871.1#62|Staphylococcus aureus
WP_217151781.1#63|Staphylococcus aureus
WP_064133630.1#64|Staphylococcus aureus
WP_053229035.1#65|Staphylococcus aureus
WP_210600641.1#66|Staphylococcus aureus
WP_179220028.1#67|Staphylococcus aureus
WP_210423004.1#68|Staphylococcus aureus
WP_216730885.1#69|Staphylococcus aureus
WP_250396404.1#70|Staphylococcus aureus
WP_198960008.1#71|Staphylococcus aureus
WP_192859173.1#72|Staphylococcus aureus
WP_207775458.1#73|Staphylococcus aureus
WP_195758717.1#74|Staphylococcus aureus
WP_064128976.1#75|Staphylococcus aureus
WP_194941929.1#76|Staphylococcus aureus
WP_180372003.1#77|Staphylococcus aureus
WP_174827527.1#78|Staphylococcus aureus
WP_203602161.1#79|Staphylococcus aureus
WP_250736785.1#80|Staphylococcus aureus
WP_181541946.1#81|Staphylococcus aureus
WP_181404032.1#82|Staphylococcus aureus
WP_181855892.1#83|Staphylococcus aureus
WP_172422660.1#84|Staphylococcus aureus
WP_001188498.1#85|Staphylococcus aureus subsp aureus LGA251
WP_020444599.1#86|Staphylococcus aureus
WP_001188490.1#87|Staphylococcus aureus
WP_180991622.1#88|Staphylococcus aureus
WP_181875225.1#89|Staphylococcus aureus
WP_281263027.1#90|Staphylococcus aureus
WP_031924988.1#91|Staphylococcus aureus WAMC6030
WP_029549877.1#92|Staphylococcus aureus
WP_233682330.1#93|Staphylococcus aureus
WP_070002257.1#94|Staphylococcus aureus
WP_206170769.1#95|Staphylococcus aureus
WP_183900032.1#96|Staphylococcus aureus
WP_181936063.1#97|Staphylococcus aureus
WP_183932991.1#98|Staphylococcus aureus
WP_054189812.1#99|Staphylococcus aureus
WP_176427358.1#100|Staphylococcus aureus
WP_001188473.1#101|Staphylococcus aureus
WP_049316015.1#102|Staphylococcus aureus
WP_205301276.1#103|Staphylococcus aureus
WP_205301163.1#104|Staphylococcus aureus
WP_181427907.1#105|Staphylococcus aureus
WP_059283842.1#106|Staphylococcus aureus
WP_218980635.1#107|Staphylococcus aureus
WP_187239734.1#108|Staphylococcus aureus
WP_224826806.1#109|Staphylococcus aureus
WP_178135736.1#110|Staphylococcus aureus
WP_205352886.1#111|Staphylococcus aureus
WP_031911903.1#112|Staphylococcus aureus USA 12
WP_217175509.1#114|Staphylococcus aureus
WP_071938114.1#115|Staphylococcus aureus
WP_216745761.1#116|Staphylococcus aureus
WP_031838317.1#117|Staphylococcus aureus VET0236R
WP_031915303.1#118|Staphylococcus aureus VET1877R
WP_178135187.1#119|Staphylococcus aureus
WP_174220641.1#120|Staphylococcus aureus
WP_258415145.1#121|Staphylococcus aureus
WP_031901081.1#122|Staphylococcus aureus MRSA 118
WP_181294570.1#123|Staphylococcus aureus
WP_031912890.1#124|Staphylococcus aureus VET0078R
WP_252570800.1#125|Staphylococcus aureus
WP_212593088.1#126|Staphylococcus aureus
WP_271291412.1#127|Staphylococcus aureus
WP_248313927.1#128|Staphylococcus aureus
WP_257553450.1#129|Staphylococcus aureus
WP_192859927.1#130|Staphylococcus aureus
WP_181935075.1#131|Staphylococcus aureus
WP_234970905.1#132|Staphylococcus aureus
WP_031911864.1#133|Staphylococcus aureus VET0070R
WP_258413198.1#134|Staphylococcus aureus
WP_172452289.1#135|Staphylococcus aureus
WP_214308888.1#136|Staphylococcus aureus
WP_249986091.1#137|Staphylococcus aureus
WP_048665175.1#138|Staphylococcus aureus
WP_069723996.1#139|Staphylococcus aureus
WP_187470623.1#141|Staphylococcus aureus
WP_248313750.1#142|Staphylococcus aureus
WP_229963790.1#144|Staphylococcus aureus
WP_187216424.1#145|Staphylococcus aureus
WP_050439799.1#146|Staphylococcus aureus 1110701127
WP_095319840.1#148|Staphylococcus aureus
WP_220674019.1#149|Staphylococcus aureus
WP_185646659.1#150|Staphylococcus aureus
WP_207771745.1#151|Staphylococcus aureus
WP_249985306.1#152|Staphylococcus aureus
WP_181450796.1#153|Staphylococcus aureus
WP_044166028.1#155|Staphylococcus aureus
WP_200641135.1#157|Staphylococcus aureus
WP_050439801.1#158|Staphylococcus aureus 1110701127
WP_220674083.1#159|Staphylococcus aureus
WP_216778170.1#160|Staphylococcus aureus
WP_180276250.1#161|Staphylococcus aureus
WP_205403790.1#162|Staphylococcus aureus
WP_172409205.1#163|Staphylococcus aureus
WP_191962784.1#164|Staphylococcus aureus
WP_046399071.1#165|Staphylococcus aureus LCT SAS
WP_254241136.1#166|Staphylococcus aureus
WP_031890355.1#167|Staphylococcus aureus M1486
WP_225549177.1#168|Staphylococcus aureus
WP_172404266.1#169|Staphylococcus aureus
WP_203228748.1#171|Staphylococcus aureus
WP_042857240.1#172|Staphylococcus aureus SA10
WP_251954985.1#173|Staphylococcus aureus
WP_172409270.1#174|Staphylococcus aureus
WP_180276053.1#175|Staphylococcus aureus
WP_224159460.1#176|Staphylococcus aureus
WP_205402336.1#177|Staphylococcus aureus
WP_146763927.1#178|Staphylococcus aureus
WP_205403868.1#179|Staphylococcus aureus
WP_191962718.1#180|Staphylococcus aureus
WP_200640834.1#181|Staphylococcus aureus
WP_031806657.1#182|Staphylococcus aureus M1070
WP_150531438.1#183|Staphylococcus aureus
WP_046472981.1#184|Staphylococcus aureus
WP_048520234.1#185|Staphylococcus aureus
WP_130118435.1#186|Staphylococcus aureus
WP_256083988.1#187|Staphylococcus aureus
