# Supplementary material for: A type VII-secreted lipase toxin with reverse domain arrangement
Source: Nat Commun. 2023 Dec 19;14:8438. doi: 10.1038/s41467-023-44221-y (PMC10730906; doi:10.1038/s41467-023-44221-y)
Supplement: Supplementary file 6 — Source Data [file 41467_2023_44221_MOESM6_ESM.zip › Tsl1 distribution raw/lplIII 1/FlaGs_output/results_operon.pdf]

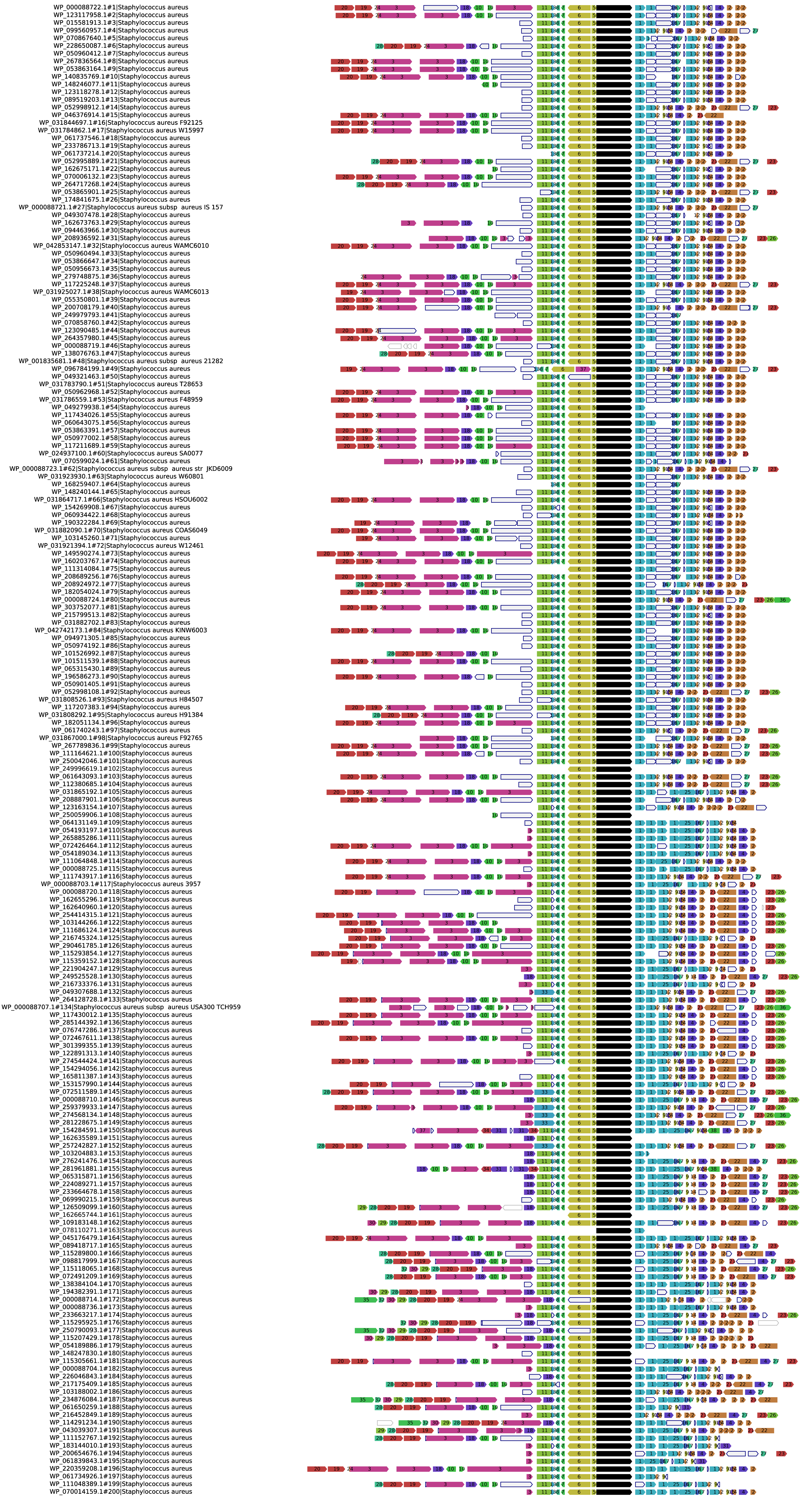
WP_000088722.1#1|Staphylococcus aureus
WP_123117958.1#2|Staphylococcus aureus
WP_015581913.1#3|Staphylococcus aureus
WP_099560957.1#4|Staphylococcus aureus
WP_070867640.1#5|Staphylococcus aureus
WP_228650087.1#6|Staphylococcus aureus
WP_050960412.1#7|Staphylococcus aureus
WP_267836564.1#8|Staphylococcus aureus
WP_053863164.1#9|Staphylococcus aureus
WP_140835769.1#10|Staphylococcus aureus
WP_148246077.1#11|Staphylococcus aureus
WP_123118278.1#12|Staphylococcus aureus
WP_089519203.1#13|Staphylococcus aureus
WP_052998912.1#14|Staphylococcus aureus
WP_046376914.1#15|Staphylococcus aureus
WP_031844697.1#16|Staphylococcus aureus F92125
WP_031784862.1#17|Staphylococcus aureus W15997
WP_061737546.1#18|Staphylococcus aureus
WP_233786713.1#19|Staphylococcus aureus
WP_061737214.1#20|Staphylococcus aureus
WP_052995889.1#21|Staphylococcus aureus
WP_162675171.1#22|Staphylococcus aureus
WP_070006132.1#23|Staphylococcus aureus
WP_264717268.1#24|Staphylococcus aureus
WP_053865901.1#25|Staphylococcus aureus
WP_174841675.1#26|Staphylococcus aureus
WP_000088721.1#27|Staphylococcus aureus subsp aureus IS 157
WP_049307478.1#28|Staphylococcus aureus
WP_162673763.1#29|Staphylococcus aureus
WP_094463966.1#30|Staphylococcus aureus
WP_208936592.1#31|Staphylococcus aureus
WP_042853147.1#32|Staphylococcus aureus WAMC6010
WP_050960494.1#33|Staphylococcus aureus
WP_053866647.1#34|Staphylococcus aureus
WP_050956673.1#35|Staphylococcus aureus
WP_279748875.1#36|Staphylococcus aureus
WP_117225248.1#37|Staphylococcus aureus
WP_031925027.1#38|Staphylococcus aureus WAMC6013
WP_055350801.1#39|Staphylococcus aureus
WP_200708179.1#40|Staphylococcus aureus
WP_249979793.1#41|Staphylococcus aureus
WP_070858760.1#42|Staphylococcus aureus
WP_123090485.1#44|Staphylococcus aureus
WP_264357980.1#45|Staphylococcus aureus
WP_000088719.1#46|Staphylococcus aureus
WP_138076763.1#47|Staphylococcus aureus
WP_001835681.1#48|Staphylococcus aureus subsp aureus 21282
WP_096784199.1#49|Staphylococcus aureus
WP_049321463.1#50|Staphylococcus aureus
WP_031783790.1#51|Staphylococcus aureus T28653
WP_050962968.1#52|Staphylococcus aureus
WP_031786559.1#53|Staphylococcus aureus F48959
WP_049279938.1#54|Staphylococcus aureus
WP_117434026.1#55|Staphylococcus aureus
WP_060643075.1#56|Staphylococcus aureus
WP_053863391.1#57|Staphylococcus aureus
WP_050977002.1#58|Staphylococcus aureus
WP_117211689.1#59|Staphylococcus aureus
WP_024937100.1#60|Staphylococcus aureus SA0077
WP_070599024.1#61|Staphylococcus aureus
WP_000088723.1#62|Staphylococcus aureus subsp aureus str JKD6009
WP_031923930.1#63|Staphylococcus aureus W60801
WP_168259407.1#64|Staphylococcus aureus
WP_148240144.1#65|Staphylococcus aureus
WP_031864717.1#66|Staphylococcus aureus HSOU6002
WP_154269908.1#67|Staphylococcus aureus
WP_060934422.1#68|Staphylococcus aureus
WP_190322284.1#69|Staphylococcus aureus
WP_031882090.1#70|Staphylococcus aureus COAS6049
WP_103145260.1#71|Staphylococcus aureus
WP_031921394.1#72|Staphylococcus aureus W12461
WP_149590274.1#73|Staphylococcus aureus
WP_160203767.1#74|Staphylococcus aureus
WP_111314084.1#75|Staphylococcus aureus
WP_208689256.1#76|Staphylococcus aureus
WP_208924972.1#77|Staphylococcus aureus
WP_182054024.1#79|Staphylococcus aureus
WP_000088724.1#80|Staphylococcus aureus
WP_303752077.1#81|Staphylococcus aureus
WP_215799513.1#82|Staphylococcus aureus
WP_031882702.1#83|Staphylococcus aureus
WP_042742173.1#84|Staphylococcus aureus KINW6003
WP_094971305.1#85|Staphylococcus aureus
WP_050974192.1#86|Staphylococcus aureus
WP_101526992.1#87|Staphylococcus aureus
WP_101511539.1#88|Staphylococcus aureus
WP_065315430.1#89|Staphylococcus aureus
WP_196586273.1#90|Staphylococcus aureus
WP_050901405.1#91|Staphylococcus aureus
WP_052998108.1#92|Staphylococcus aureus
WP_031808526.1#93|Staphylococcus aureus H84507
WP_117207383.1#94|Staphylococcus aureus
WP_031808292.1#95|Staphylococcus aureus H91384
WP_182051134.1#96|Staphylococcus aureus
WP_061740243.1#97|Staphylococcus aureus
WP_031867000.1#98|Staphylococcus aureus F92765
WP_267789836.1#99|Staphylococcus aureus
WP_111164621.1#100|Staphylococcus aureus
WP_250042046.1#101|Staphylococcus aureus
WP_249996619.1#102|Staphylococcus aureus
WP_061643093.1#103|Staphylococcus aureus
WP_112380685.1#104|Staphylococcus aureus
WP_031865192.1#105|Staphylococcus aureus
WP_208887901.1#106|Staphylococcus aureus
WP_123163154.1#107|Staphylococcus aureus
WP_250059906.1#108|Staphylococcus aureus
WP_064131149.1#109|Staphylococcus aureus
WP_054193197.1#110|Staphylococcus aureus
WP_265885286.1#111|Staphylococcus aureus
WP_072426464.1#112|Staphylococcus aureus
WP_054189034.1#113|Staphylococcus aureus
WP_111064848.1#114|Staphylococcus aureus
WP_000088725.1#115|Staphylococcus aureus
WP_111743917.1#116|Staphylococcus aureus
WP_000088703.1#117|Staphylococcus aureus 3957
WP_000088720.1#118|Staphylococcus aureus
WP_162655296.1#119|Staphylococcus aureus
WP_162640960.1#120|Staphylococcus aureus
WP_254414315.1#121|Staphylococcus aureus
WP_103144266.1#122|Staphylococcus aureus
WP_111686124.1#124|Staphylococcus aureus
WP_216745324.1#125|Staphylococcus aureus
WP_290461785.1#126|Staphylococcus aureus
WP_115293854.1#127|Staphylococcus aureus
WP_115359152.1#128|Staphylococcus aureus
WP_221904247.1#129|Staphylococcus aureus
WP_249525528.1#130|Staphylococcus aureus
WP_216733376.1#131|Staphylococcus aureus
WP_049307688.1#132|Staphylococcus aureus
WP_264128728.1#133|Staphylococcus aureus
WP_000088707.1#134|Staphylococcus aureus subsp aureus USA300 TCH959
WP_117430012.1#135|Staphylococcus aureus
WP_285144392.1#136|Staphylococcus aureus
WP_076747286.1#137|Staphylococcus aureus
WP_072467611.1#138|Staphylococcus aureus
WP_301399355.1#139|Staphylococcus aureus
WP_122891313.1#140|Staphylococcus aureus
WP_274544424.1#141|Staphylococcus aureus
WP_154294056.1#142|Staphylococcus aureus
WP_165811387.1#143|Staphylococcus aureus
WP_153157990.1#144|Staphylococcus aureus
WP_072511589.1#145|Staphylococcus aureus
WP_000088710.1#146|Staphylococcus aureus
WP_259379933.1#147|Staphylococcus aureus
WP_274568134.1#148|Staphylococcus aureus
WP_281228675.1#149|Staphylococcus aureus
WP_154284591.1#150|Staphylococcus aureus
WP_162635589.1#151|Staphylococcus aureus
WP_257242827.1#152|Staphylococcus aureus
WP_103204883.1#153|Staphylococcus aureus
WP_276241476.1#154|Staphylococcus aureus
WP_281961881.1#155|Staphylococcus aureus
WP_065315871.1#156|Staphylococcus aureus
WP_224089271.1#157|Staphylococcus aureus
WP_233664678.1#158|Staphylococcus aureus
WP_069990215.1#159|Staphylococcus aureus
WP_126509099.1#160|Staphylococcus aureus
WP_162665744.1#161|Staphylococcus aureus
WP_109183148.1#162|Staphylococcus aureus
WP_078110271.1#163|Staphylococcus aureus
WP_045176479.1#164|Staphylococcus aureus
WP_089418717.1#165|Staphylococcus aureus
WP_115289800.1#166|Staphylococcus aureus
WP_098817999.1#167|Staphylococcus aureus
WP_115118065.1#168|Staphylococcus aureus
WP_072491209.1#169|Staphylococcus aureus
WP_138384104.1#170|Staphylococcus aureus
WP_194382391.1#171|Staphylococcus aureus
WP_000088714.1#172|Staphylococcus aureus
WP_000088736.1#173|Staphylococcus aureus
WP_233663217.1#174|Staphylococcus aureus
WP_115295925.1#176|Staphylococcus aureus
WP_250790093.1#177|Staphylococcus aureus
WP_115207429.1#178|Staphylococcus aureus
WP_054189886.1#179|Staphylococcus aureus
WP_148247830.1#180|Staphylococcus aureus
WP_115305661.1#181|Staphylococcus aureus
WP_000088704.1#182|Staphylococcus aureus
WP_226046843.1#184|Staphylococcus aureus
WP_217175409.1#185|Staphylococcus aureus
WP_103188002.1#186|Staphylococcus aureus
WP_234876084.1#187|Staphylococcus aureus
WP_061650259.1#188|Staphylococcus aureus
WP_216452849.1#189|Staphylococcus aureus
WP_114291234.1#190|Staphylococcus aureus
WP_043039307.1#191|Staphylococcus aureus
WP_111152767.1#192|Staphylococcus aureus
WP_183144010.1#193|Staphylococcus aureus
WP_200654676.1#194|Staphylococcus aureus
WP_061839843.1#195|Staphylococcus aureus
WP_220359208.1#196|Staphylococcus aureus
WP_061734926.1#197|Staphylococcus aureus
WP_111048389.1#199|Staphylococcus aureus
WP_070014159.1#200|Staphylococcus aureus
