# Supplementary material for: A type VII-secreted lipase toxin with reverse domain arrangement
Source: Nat Commun. 2023 Dec 19;14:8438. doi: 10.1038/s41467-023-44221-y (PMC10730906; doi:10.1038/s41467-023-44221-y)
Supplement: Supplementary file 6 — Source Data [file 41467_2023_44221_MOESM6_ESM.zip › Tsl1 distribution raw/lplIII 3/FlaGs_output/results_TreeOrder_output.pdf]

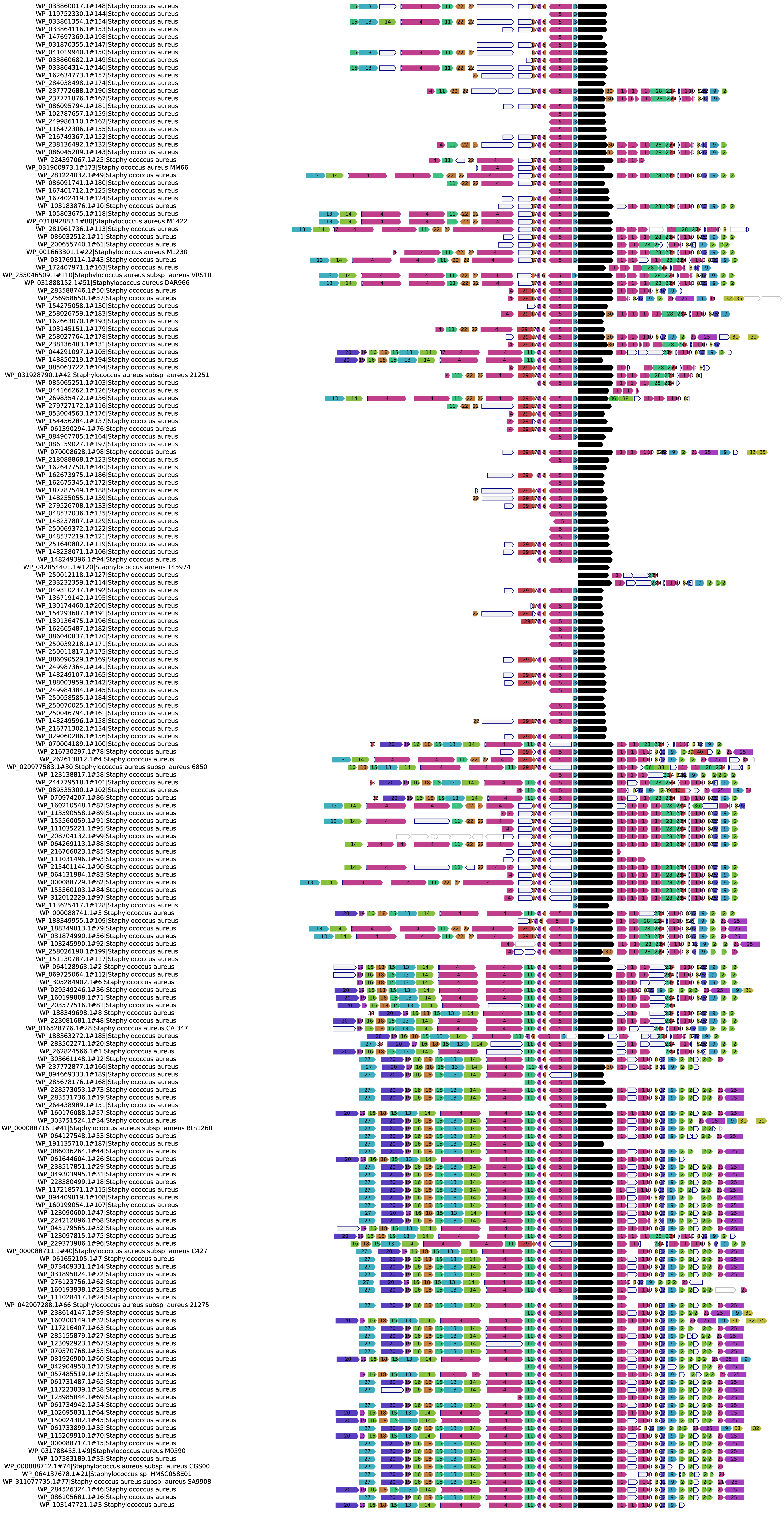
WP_033860017.1#148|Staphylococcus aureus
WP_119752330.1#144|Staphylococcus aureus
WP_033861354.1#154|Staphylococcus aureus
WP_033864116.1#153|Staphylococcus aureus
WP_147697369.1#198|Staphylococcus aureus
WP_031870355.1#147|Staphylococcus aureus
WP_041019940.1#150|Staphylococcus aureus
WP_033860682.1#149|Staphylococcus aureus
WP_033864314.1#146|Staphylococcus aureus
WP_162634773.1#157|Staphylococcus aureus
WP_284038498.1#174|Staphylococcus aureus
WP_237772688.1#190|Staphylococcus aureus
WP_237771876.1#167|Staphylococcus aureus
WP_086095794.1#181|Staphylococcus aureus
WP_102787657.1#159|Staphylococcus aureus
WP_249986110.1#162|Staphylococcus aureus
WP_116472306.1#155|Staphylococcus aureus
WP_216749367.1#152|Staphylococcus aureus
WP_238136492.1#132|Staphylococcus aureus
WP_086045209.1#143|Staphylococcus aureus
WP_224397067.1#25|Staphylococcus aureus
WP_031900973.1#173|Staphylococcus aureus MM66
WP_281224032.1#49|Staphylococcus aureus
WP_086091741.1#180|Staphylococcus aureus
WP_167401712.1#125|Staphylococcus aureus
WP_167402419.1#124|Staphylococcus aureus
WP_103183876.1#10|Staphylococcus aureus
WP_105803675.1#118|Staphylococcus aureus
WP_031892883.1#80|Staphylococcus aureus M1422
WP_281961736.1#113|Staphylococcus aureus
WP_086032512.1#11|Staphylococcus aureus
WP_200655740.1#61|Staphylococcus aureus
WP_001663301.1#22|Staphylococcus aureus M1230
WP_031769114.1#43|Staphylococcus aureus
WP_172407971.1#163|Staphylococcus aureus
WP_235046509.1#110|Staphylococcus aureus subsp aureus VRS10
WP_031888152.1#51|Staphylococcus aureus DAR966
WP_283588746.1#50|Staphylococcus aureus
WP_256958650.1#37|Staphylococcus aureus
WP_154275058.1#130|Staphylococcus aureus
WP_258026759.1#183|Staphylococcus aureus
WP_162663070.1#193|Staphylococcus aureus
WP_103145151.1#179|Staphylococcus aureus
WP_258027764.1#178|Staphylococcus aureus
WP_238136483.1#131|Staphylococcus aureus
WP_044291097.1#105|Staphylococcus aureus
WP_148850219.1#194|Staphylococcus aureus
WP_085063722.1#104|Staphylococcus aureus
WP_031928790.1#42|Staphylococcus aureus subsp aureus 21251
WP_085065251.1#103|Staphylococcus aureus
WP_044166262.1#126|Staphylococcus aureus
WP_269835472.1#136|Staphylococcus aureus
WP_279727172.1#116|Staphylococcus aureus
WP_053004563.1#176|Staphylococcus aureus
WP_154456284.1#137|Staphylococcus aureus
WP_061390294.1#76|Staphylococcus aureus
WP_084967705.1#164|Staphylococcus aureus
WP_086159027.1#197|Staphylococcus aureus
WP_070008628.1#98|Staphylococcus aureus
WP_218088868.1#123|Staphylococcus aureus
WP_162647750.1#140|Staphylococcus aureus
WP_162673975.1#186|Staphylococcus aureus
WP_162675345.1#172|Staphylococcus aureus
WP_187787549.1#188|Staphylococcus aureus
WP_148255055.1#139|Staphylococcus aureus
WP_279526708.1#133|Staphylococcus aureus
WP_048537036.1#135|Staphylococcus aureus
WP_148237807.1#129|Staphylococcus aureus
WP_250069372.1#122|Staphylococcus aureus
WP_048537219.1#121|Staphylococcus aureus
WP_251640802.1#119|Staphylococcus aureus
WP_148238071.1#106|Staphylococcus aureus
WP_148249396.1#94|Staphylococcus aureus
WP_042854401.1#120|Staphylococcus aureus T45974
WP_250012118.1#127|Staphylococcus aureus
WP_233232359.1#114|Staphylococcus aureus
WP_049310237.1#192|Staphylococcus aureus
WP_136719142.1#195|Staphylococcus aureus
WP_130174460.1#200|Staphylococcus aureus
WP_154293607.1#191|Staphylococcus aureus
WP_130136475.1#196|Staphylococcus aureus
WP_162665487.1#182|Staphylococcus aureus
WP_086040837.1#170|Staphylococcus aureus
WP_250039218.1#171|Staphylococcus aureus
WP_250011817.1#175|Staphylococcus aureus
WP_086090529.1#169|Staphylococcus aureus
WP_249987364.1#141|Staphylococcus aureus
WP_148249107.1#165|Staphylococcus aureus
WP_188003959.1#142|Staphylococcus aureus
WP_249984384.1#145|Staphylococcus aureus
WP_250058585.1#184|Staphylococcus aureus
WP_250070025.1#160|Staphylococcus aureus
WP_250046794.1#161|Staphylococcus aureus
WP_148259596.1#158|Staphylococcus aureus
WP_216771302.1#134|Staphylococcus aureus
WP_029060286.1#156|Staphylococcus aureus
WP_070004189.1#100|Staphylococcus aureus
WP_216730297.1#78|Staphylococcus aureus
WP_262613812.1#4|Staphylococcus aureus
WP_020977583.1#30|Staphylococcus aureus subsp aureus 6850
WP_123138817.1#58|Staphylococcus aureus
WP_244779518.1#101|Staphylococcus aureus
WP_089535300.1#102|Staphylococcus aureus
WP_070974207.1#86|Staphylococcus aureus
WP_160210548.1#87|Staphylococcus aureus
WP_113590558.1#89|Staphylococcus aureus
WP_155560059.1#91|Staphylococcus aureus
WP_111035221.1#95|Staphylococcus aureus
WP_208704132.1#99|Staphylococcus aureus
WP_064269113.1#88|Staphylococcus aureus
WP_216766023.1#85|Staphylococcus aureus
WP_111031496.1#93|Staphylococcus aureus
WP_215401144.1#90|Staphylococcus aureus
WP_064131984.1#83|Staphylococcus aureus
WP_000088729.1#82|Staphylococcus aureus
WP_155560103.1#84|Staphylococcus aureus
WP_312012229.1#97|Staphylococcus aureus
WP_113625417.1#128|Staphylococcus aureus
WP_000088741.1#5|Staphylococcus aureus
WP_188349955.1#109|Staphylococcus aureus
WP_188349813.1#79|Staphylococcus aureus
WP_031874990.1#56|Staphylococcus aureus
WP_103245990.1#92|Staphylococcus aureus
WP_258026190.1#199|Staphylococcus aureus
WP_151130787.1#117|Staphylococcus aureus
WP_064128963.1#2|Staphylococcus aureus
WP_069725064.1#112|Staphylococcus aureus
WP_305284902.1#6|Staphylococcus aureus
WP_029549246.1#36|Staphylococcus aureus
WP_160199808.1#71|Staphylococcus aureus
WP_203577516.1#81|Staphylococcus aureus
WP_188349698.1#8|Staphylococcus aureus
WP_223081681.1#48|Staphylococcus aureus
WP_016528776.1#28|Staphylococcus aureus CA 347
WP_188363272.1#185|Staphylococcus aureus
WP_283502271.1#20|Staphylococcus aureus
WP_262824566.1#1|Staphylococcus aureus
WP_303661148.1#12|Staphylococcus aureus
WP_237772877.1#166|Staphylococcus aureus
WP_094669333.1#189|Staphylococcus aureus
WP_285678176.1#168|Staphylococcus aureus
WP_228573053.1#73|Staphylococcus aureus
WP_283531736.1#19|Staphylococcus aureus
WP_264438989.1#151|Staphylococcus aureus
WP_160176088.1#57|Staphylococcus aureus
WP_303751524.1#34|Staphylococcus aureus
WP_000088716.1#41|Staphylococcus aureus subsp aureus Btn1260
WP_064127548.1#53|Staphylococcus aureus
WP_191135710.1#187|Staphylococcus aureus
WP_086036264.1#44|Staphylococcus aureus
WP_061644604.1#26|Staphylococcus aureus
WP_238517851.1#29|Staphylococcus aureus
WP_049303995.1#31|Staphylococcus aureus
WP_228580499.1#18|Staphylococcus aureus
WP_117218571.1#115|Staphylococcus aureus
WP_094409819.1#108|Staphylococcus aureus
WP_160199054.1#107|Staphylococcus aureus
WP_123090600.1#47|Staphylococcus aureus
WP_224212096.1#68|Staphylococcus aureus
WP_045179565.1#52|Staphylococcus aureus
WP_123097815.1#75|Staphylococcus aureus
WP_229373986.1#96|Staphylococcus aureus
WP_000088711.1#40|Staphylococcus aureus subsp aureus C427
WP_061652105.1#7|Staphylococcus aureus
WP_073409331.1#14|Staphylococcus aureus
WP_031895024.1#72|Staphylococcus aureus
WP_276123756.1#62|Staphylococcus aureus
WP_160193938.1#23|Staphylococcus aureus
WP_111028417.1#24|Staphylococcus aureus
WP_042907288.1#66|Staphylococcus aureus subsp aureus 21275
WP_238614147.1#39|Staphylococcus aureus
WP_160200149.1#32|Staphylococcus aureus
WP_117216407.1#63|Staphylococcus aureus
WP_285155879.1#27|Staphylococcus aureus
WP_123092923.1#67|Staphylococcus aureus
WP_070570768.1#55|Staphylococcus aureus
WP_031926900.1#60|Staphylococcus aureus
WP_042904950.1#17|Staphylococcus aureus
WP_057485519.1#13|Staphylococcus aureus
WP_061731487.1#65|Staphylococcus aureus
WP_117223839.1#38|Staphylococcus aureus
WP_123985844.1#69|Staphylococcus aureus
WP_061734942.1#54|Staphylococcus aureus
WP_102695831.1#64|Staphylococcus aureus
WP_150024302.1#45|Staphylococcus aureus
WP_061733899.1#35|Staphylococcus aureus
WP_115209910.1#70|Staphylococcus aureus
WP_000088717.1#15|Staphylococcus aureus
WP_031788453.1#9|Staphylococcus aureus M0590
WP_107383189.1#33|Staphylococcus aureus
WP_000088712.1#74|Staphylococcus aureus subsp aureus CGS00
WP_064137678.1#21|Staphylococcus sp HMSC058E01
WP_311077735.1#77|Staphylococcus aureus subsp aureus SA9908
WP_284526324.1#46|Staphylococcus aureus
WP_086105681.1#16|Staphylococcus aureus
WP_103147721.1#3|Staphylococcus aureus
