# Supplementary material for: A type VII-secreted lipase toxin with reverse domain arrangement
Source: Nat Commun. 2023 Dec 19;14:8438. doi: 10.1038/s41467-023-44221-y (PMC10730906; doi:10.1038/s41467-023-44221-y)
Supplement: Supplementary file 6 — Source Data [file 41467_2023_44221_MOESM6_ESM.zip › Tsl1 distribution raw/lplIII 4/FlaGs_output/results_operon.pdf]

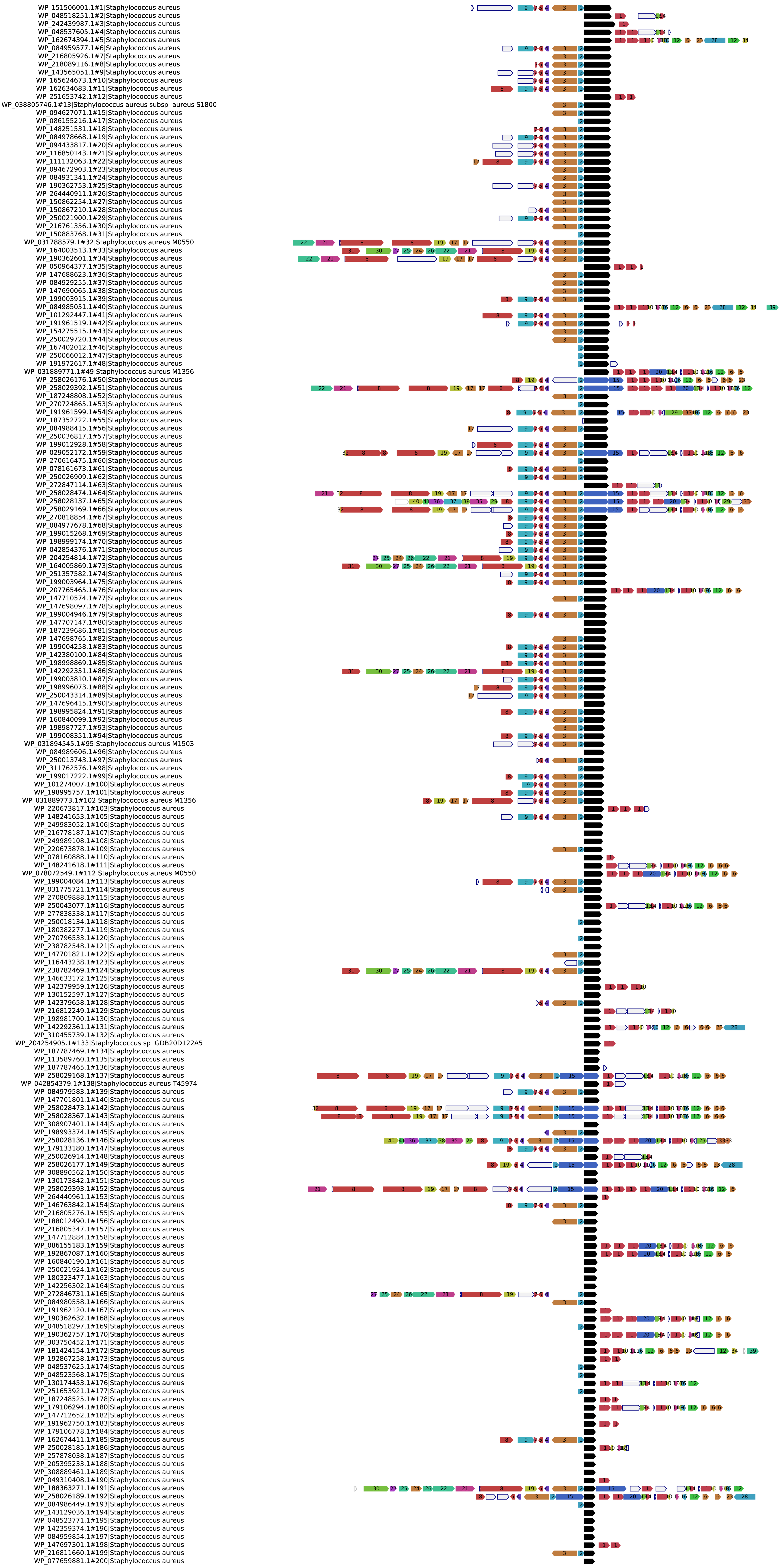
WP_151506001.1#1|Staphylococcus aureus
WP_048518251.1#2|Staphylococcus aureus
WP_242439987.1#3|Staphylococcus aureus
WP_048537605.1#4|Staphylococcus aureus
WP_162674394.1#5|Staphylococcus aureus
WP_084959577.1#6|Staphylococcus aureus
WP_216805926.1#7|Staphylococcus aureus
WP_218089116.1#8|Staphylococcus aureus
WP_143565051.1#9|Staphylococcus aureus
WP_165624673.1#10|Staphylococcus aureus
WP_162634683.1#11|Staphylococcus aureus
WP_251653742.1#12|Staphylococcus aureus
WP_038805746.1#13|Staphylococcus aureus subsp aureus S1800
WP_094627071.1#15|Staphylococcus aureus
WP_086155216.1#17|Staphylococcus aureus
WP_148251531.1#18|Staphylococcus aureus
WP_084978668.1#19|Staphylococcus aureus
WP_094433817.1#20|Staphylococcus aureus
WP_116850143.1#21|Staphylococcus aureus
WP_111132063.1#22|Staphylococcus aureus
WP_094672903.1#23|Staphylococcus aureus
WP_084931341.1#24|Staphylococcus aureus
WP_190362753.1#25|Staphylococcus aureus
WP_264440911.1#26|Staphylococcus aureus
WP_150862254.1#27|Staphylococcus aureus
WP_150867210.1#28|Staphylococcus aureus
WP_250021900.1#29|Staphylococcus aureus
WP_216761356.1#30|Staphylococcus aureus
WP_150883768.1#31|Staphylococcus aureus
WP_031788579.1#32|Staphylococcus aureus M0550
WP_164003513.1#33|Staphylococcus aureus
WP_190362601.1#34|Staphylococcus aureus
WP_050964377.1#35|Staphylococcus aureus
WP_147688623.1#36|Staphylococcus aureus
WP_084929255.1#37|Staphylococcus aureus
WP_147690065.1#38|Staphylococcus aureus
WP_199003915.1#39|Staphylococcus aureus
WP_084985051.1#40|Staphylococcus aureus
WP_101292447.1#41|Staphylococcus aureus
WP_191961519.1#42|Staphylococcus aureus
WP_154275515.1#43|Staphylococcus aureus
WP_250029720.1#44|Staphylococcus aureus
WP_167402012.1#46|Staphylococcus aureus
WP_250066012.1#47|Staphylococcus aureus
WP_191972617.1#48|Staphylococcus aureus
WP_031889771.1#49|Staphylococcus aureus M1356
WP_258026176.1#50|Staphylococcus aureus
WP_258029392.1#51|Staphylococcus aureus
WP_187248808.1#52|Staphylococcus aureus
WP_270724865.1#53|Staphylococcus aureus
WP_191961599.1#54|Staphylococcus aureus
WP_187352722.1#55|Staphylococcus aureus
WP_084988415.1#56|Staphylococcus aureus
WP_250036817.1#57|Staphylococcus aureus
WP_199012928.1#58|Staphylococcus aureus
WP_029052172.1#59|Staphylococcus aureus
WP_270616475.1#60|Staphylococcus aureus
WP_078161673.1#61|Staphylococcus aureus
WP_250026909.1#62|Staphylococcus aureus
WP_272847114.1#63|Staphylococcus aureus
WP_258028474.1#64|Staphylococcus aureus
WP_258028137.1#65|Staphylococcus aureus
WP_258029169.1#66|Staphylococcus aureus
WP_270818854.1#67|Staphylococcus aureus
WP_084977678.1#68|Staphylococcus aureus
WP_199015268.1#69|Staphylococcus aureus
WP_198999174.1#70|Staphylococcus aureus
WP_042854376.1#71|Staphylococcus aureus
WP_204254814.1#72|Staphylococcus aureus
WP_164005869.1#73|Staphylococcus aureus
WP_251357582.1#74|Staphylococcus aureus
WP_199003964.1#75|Staphylococcus aureus
WP_207765465.1#76|Staphylococcus aureus
WP_147710574.1#77|Staphylococcus aureus
WP_147698097.1#78|Staphylococcus aureus
WP_199004946.1#79|Staphylococcus aureus
WP_147707147.1#80|Staphylococcus aureus
WP_187239686.1#81|Staphylococcus aureus
WP_147698765.1#82|Staphylococcus aureus
WP_199004258.1#83|Staphylococcus aureus
WP_142380100.1#84|Staphylococcus aureus
WP_198998869.1#85|Staphylococcus aureus
WP_142292351.1#86|Staphylococcus aureus
WP_199003810.1#87|Staphylococcus aureus
WP_198996073.1#88|Staphylococcus aureus
WP_250043314.1#89|Staphylococcus aureus
WP_147696415.1#90|Staphylococcus aureus
WP_198995823.1#91|Staphylococcus aureus
WP_160840099.1#92|Staphylococcus aureus
WP_198987727.1#93|Staphylococcus aureus
WP_199008351.1#94|Staphylococcus aureus
WP_031894545.1#95|Staphylococcus aureus M1503
WP_084989606.1#96|Staphylococcus aureus
WP_250013743.1#97|Staphylococcus aureus
WP_311762576.1#98|Staphylococcus aureus
WP_199017222.1#99|Staphylococcus aureus
WP_101274007.1#100|Staphylococcus aureus
WP_198995757.1#101|Staphylococcus aureus
WP_031889773.1#102|Staphylococcus aureus M1356
WP_220673817.1#103|Staphylococcus aureus
WP_148241653.1#105|Staphylococcus aureus
WP_249983052.1#106|Staphylococcus aureus
WP_216778187.1#107|Staphylococcus aureus
WP_249989108.1#108|Staphylococcus aureus
WP_220673878.1#109|Staphylococcus aureus
WP_078160888.1#110|Staphylococcus aureus
WP_148241618.1#111|Staphylococcus aureus
WP_078072549.1#112|Staphylococcus aureus M0550
WP_199004084.1#113|Staphylococcus aureus
WP_031775721.1#114|Staphylococcus aureus
WP_270809888.1#115|Staphylococcus aureus
WP_250043077.1#116|Staphylococcus aureus
WP_277838338.1#117|Staphylococcus aureus
WP_250018134.1#118|Staphylococcus aureus
WP_180382277.1#119|Staphylococcus aureus
WP_270796533.1#120|Staphylococcus aureus
WP_238782548.1#121|Staphylococcus aureus
WP_147701821.1#122|Staphylococcus aureus
WP_116443238.1#123|Staphylococcus aureus
WP_238782469.1#124|Staphylococcus aureus
WP_146633172.1#125|Staphylococcus aureus
WP_142379959.1#126|Staphylococcus aureus
WP_130152597.1#127|Staphylococcus aureus
WP_142379658.1#128|Staphylococcus aureus
WP_216812249.1#129|Staphylococcus aureus
WP_198981700.1#130|Staphylococcus aureus
WP_142292361.1#131|Staphylococcus aureus
WP_310455739.1#132|Staphylococcus aureus
WP_204254905.1#133|Staphylococcus sp GDB20D122A5
WP_187787469.1#134|Staphylococcus aureus
WP_113589760.1#135|Staphylococcus aureus
WP_187787465.1#136|Staphylococcus aureus
WP_258029168.1#137|Staphylococcus aureus
WP_042854379.1#138|Staphylococcus aureus T45974
WP_084979583.1#139|Staphylococcus aureus
WP_147701801.1#140|Staphylococcus aureus
WP_258028473.1#142|Staphylococcus aureus
WP_258028367.1#143|Staphylococcus aureus
WP_308907401.1#144|Staphylococcus aureus
WP_198993374.1#145|Staphylococcus aureus
WP_258028136.1#146|Staphylococcus aureus
WP_179133180.1#147|Staphylococcus aureus
WP_250026914.1#148|Staphylococcus aureus
WP_258026177.1#149|Staphylococcus aureus
WP_308890562.1#150|Staphylococcus aureus
WP_130173842.1#151|Staphylococcus aureus
WP_258029393.1#152|Staphylococcus aureus
WP_264440961.1#153|Staphylococcus aureus
WP_146763842.1#154|Staphylococcus aureus
WP_216805276.1#155|Staphylococcus aureus
WP_188012490.1#156|Staphylococcus aureus
WP_216805347.1#157|Staphylococcus aureus
WP_147712884.1#158|Staphylococcus aureus
WP_086155183.1#159|Staphylococcus aureus
WP_192867087.1#160|Staphylococcus aureus
WP_160840190.1#161|Staphylococcus aureus
WP_250021924.1#162|Staphylococcus aureus
WP_180323477.1#163|Staphylococcus aureus
WP_142256302.1#164|Staphylococcus aureus
WP_272846731.1#165|Staphylococcus aureus
WP_084980558.1#166|Staphylococcus aureus
WP_191962120.1#167|Staphylococcus aureus
WP_190362632.1#168|Staphylococcus aureus
WP_048518297.1#169|Staphylococcus aureus
WP_190362757.1#170|Staphylococcus aureus
WP_303750452.1#171|Staphylococcus aureus
WP_181424154.1#172|Staphylococcus aureus
WP_192867258.1#173|Staphylococcus aureus
WP_048537625.1#174|Staphylococcus aureus
WP_048523568.1#175|Staphylococcus aureus
WP_130174453.1#176|Staphylococcus aureus
WP_251653921.1#177|Staphylococcus aureus
WP_187248525.1#178|Staphylococcus aureus
WP_179106294.1#180|Staphylococcus aureus
WP_147712652.1#182|Staphylococcus aureus
WP_191962750.1#183|Staphylococcus aureus
WP_179106778.1#184|Staphylococcus aureus
WP_162674411.1#185|Staphylococcus aureus
WP_250028185.1#186|Staphylococcus aureus
WP_257878038.1#187|Staphylococcus aureus
WP_205395233.1#188|Staphylococcus aureus
WP_308889461.1#189|Staphylococcus aureus
WP_049310408.1#190|Staphylococcus aureus
WP_188363271.1#191|Staphylococcus aureus
WP_258026189.1#192|Staphylococcus aureus
WP_084986449.1#193|Staphylococcus aureus
WP_143129036.1#194|Staphylococcus aureus
WP_048523771.1#195|Staphylococcus aureus
WP_142359374.1#196|Staphylococcus aureus
WP_084959854.1#197|Staphylococcus aureus
WP_147697301.1#198|Staphylococcus aureus
WP_216811660.1#199|Staphylococcus aureus
WP_077659881.1#200|Staphylococcus aureus
