# Supplementary figures and images for: A type VII-secreted lipase toxin with reverse domain arrangement
Source: Nat Commun. 2023 Dec 19;14:8438. doi: 10.1038/s41467-023-44221-y (PMC10730906; doi:10.1038/s41467-023-44221-y)

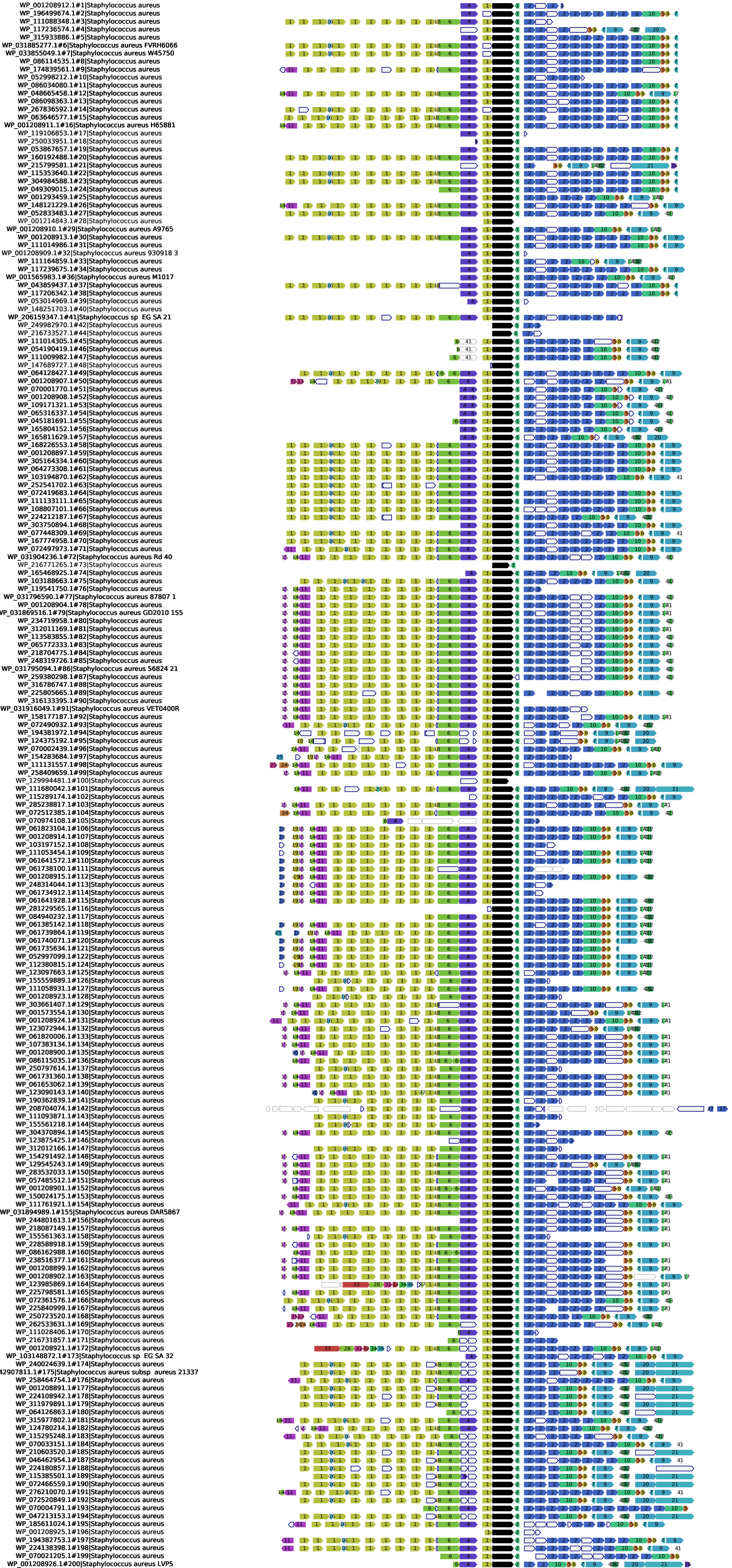

Supplement: Supplementary file 6 — Source Data [file 41467_2023_44221_MOESM6_ESM.zip › Tsl1 distribution raw/lpl0 1/FlaGs_output/results_operon.pdf]

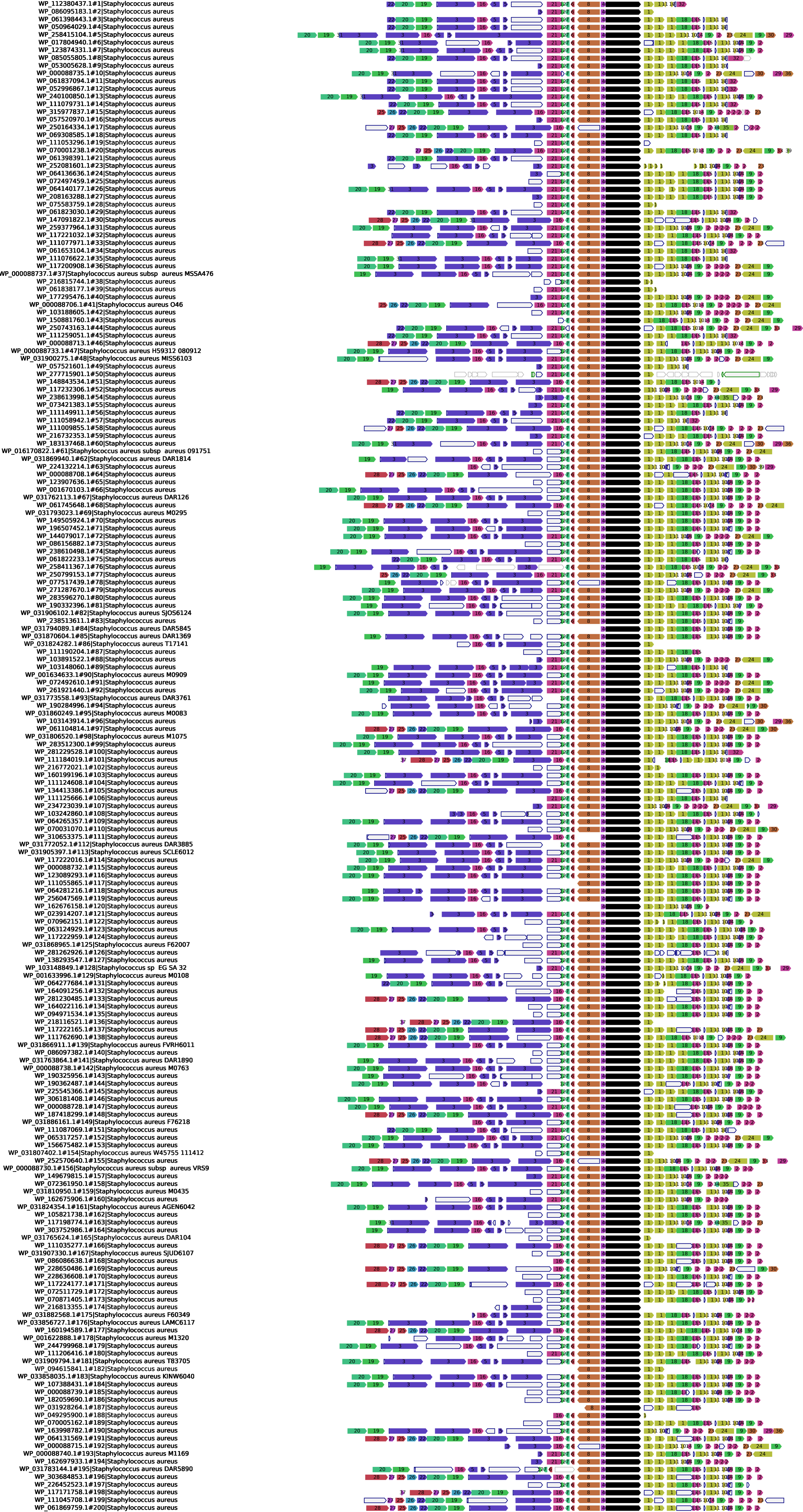

Supplement: Supplementary file 6 — Source Data [file 41467_2023_44221_MOESM6_ESM.zip › Tsl1 distribution raw/lplIII 2/FlaGs_output/results_operon.pdf]

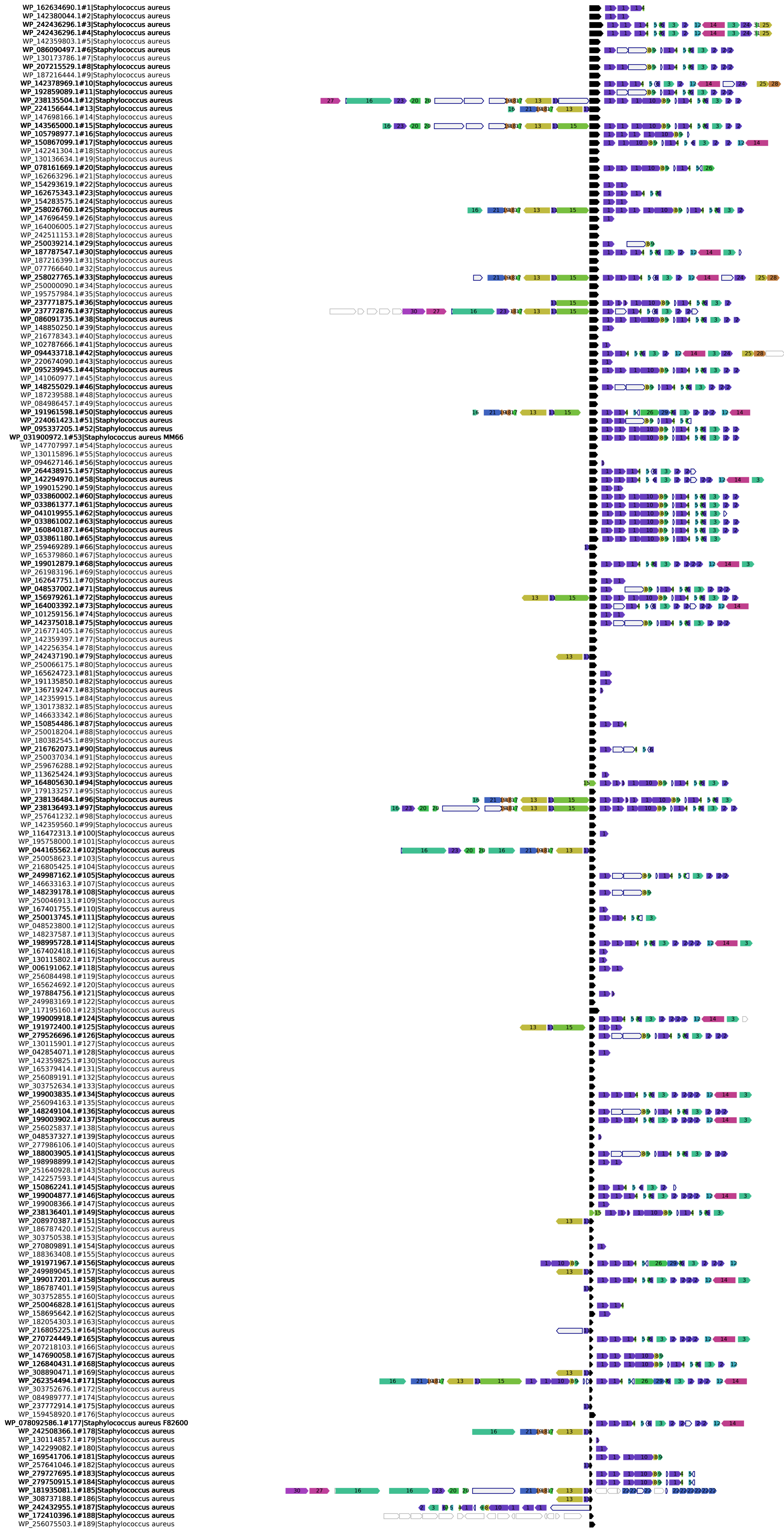

Supplement: Supplementary file 6 — Source Data [file 41467_2023_44221_MOESM6_ESM.zip › Tsl1 distribution raw/lplIII 5/FlaGs_output/results_operon.pdf]
